# Deep learning to predict cardiovascular mortality from aortic disease in heavy smokers

**DOI:** 10.1038/s44325-024-00029-3

**Published:** 2024-11-06

**Authors:** Alexander Rau, Lea Michel, Ben Wilhelm, Vineet K. Raghu, Marco Reisert, Matthias Jung, Elias Kellner, Christopher L. Schlett, Hugo J. W. L. Aerts, Michael T. Lu, Fabian Bamberg, Jakob Weiss

**Affiliations:** 1 Department of Diagnostic and Interventional Radiology, Medical Center—University of Freiburg, Faculty of Medicine, University of Freiburg, Freiburg, Germany; 2 Department of Neuroradiology, Medical Center—University of Freiburg, Faculty of Medicine, University of Freiburg, Freiburg, Germany; 3 Cardiovascular Imaging Research Center (CIRC), Department of Radiology, Massachusetts General Hospital & Harvard Medical School, Boston, MA USA; 4 Artificial Intelligence in Medicine (AIM) Program, Mass General Brigham, Harvard Medical School, Boston, MA USA; 5 Medical Physics, Department of Diagnostic and Interventional Radiology, Medical Center—University of Freiburg, Faculty of Medicine, University of Freiburg, Freiburg, Germany; 6 Department of Stereotactic and Functional Neurosurgery, Medical Center—University of Freiburg, Faculty of Medicine, University of Freiburg, Freiburg, Germany; 7 Department of Radiation Oncology, Dana-Farber Cancer Institute, Brigham and Women’s Hospital, Harvard Medical School, Boston, MA USA; 8 Department of Radiology and Nuclear Medicine, CARIM & GROW, Maastricht University, Maastricht, The Netherlands

**Keywords:** Cardiology, Cardiovascular diseases

## Abstract

Aortic angiopathy is a common manifestation of cardiovascular disease (CVD) and may serve as a surrogate marker of CVD burden. While the maximum aortic diameter is the primary prognostic measure, the potential of other features to improve risk prediction remains uncertain. This study developed a deep learning framework to automatically quantify thoracic aortic disease features and assessed their prognostic value in predicting CVD mortality among heavy smokers. Using non-contrast chest CTs from the National Lung Screening Trial (NLST), aortic features quantified included maximum diameter, volume, and calcification burden. Among 24,770 participants, 440 CVD deaths occurred over a mean 6.3-year follow-up. Aortic calcifications and volume were independently associated with CVD mortality, even after adjusting for traditional risk factors and coronary artery calcifications. These findings suggest that deep learning-derived aortic features could improve CVD risk prediction in high-risk populations, enabling more personalized prevention strategies.

## Introduction

Cardiovascular disease (CVD) remains the leading cause of death, accounting for up to 30% in the United States^1^. Extensive research over the past decades has helped to identify and understand the role of various modifiable cardiovascular risk factors (e.g., hypertension and blood lipids), which has led to the development of risk models (e.g Framingham risk score or atherosclerotic cardiovascular disease (ASCVD) risk score) to support CVD risk estimation and preventive strategies^2^. Yet, because the value of these risk models for prognostication remains limited there is an ongoing need to reliably identify those with the highest benefit from personalized prevention to further reduce morbidity and mortality^2^.

In this context coronary artery calcium scanning has gained increasing importance to improve risk assessment as it can unveil subclinical vascular changes preceding major adverse cardiovascular events^3–5^ and was therefore included in the latest American College of Cardiology/American Heart Association guidelines on the primary prevention of cardiovascular disease^6^. While coronary artery calcium assessment requires standardized scanning and post-processing algorithms^7^, there is a growing body of research suggesting that potentially relevant prognostic imaging information can be quantified opportunistically on imaging studies acquired for various indications^8–10^. In addition, although coronary artery calcium is widely accepted and one of the strongest predictors for cardiovascular events^7,11^, there is increasing evidence that the aorta, the largest vessel of the vascular system, may serve as a surrogate reflecting an overall arteriosclerotic vascular burden that carries prognostic information to estimate cardiovascular risk which has been shown for calcifications of the thoracic and abdominal aorta^12–14^.

Currently, the maximum aortic diameter is the measure most frequently used in clinical practice to objectify the extent of aortic alterations due to time and equipment constraints. Other potentially relevant findings, such as aortic volume or calcifications go unnoticed although recent research has discovered them as promising biomarkers with prognostic value^12,13,15^. Yet, it remains unknown whether they carry incremental prognostic value compared to traditional risk factors and coronary artery calcium in large-scale population-wide analysis. With novel deep learning methods, a form of artificial intelligence, it has now become feasible to automatically quantify these features in cross-sectional imaging studies with the potential to improve risk assessment among individuals^16–19^.

Here, we propose a deep learning framework that automatically quantifies features of thoracic aortic disease on non-contrast chest computed tomography (CT) scans. We hypothesized that this allows for opportunistic identification of individuals at increased risk of cardiovascular mortality beyond the maximum aortic diameter, traditional cardiovascular risk factors, and coronary artery calcifications (CAC). The prognostic value of the framework was tested in 24,770 individuals of the National Lung Screening Trial (NLST), who are at increased risk for cardiovascular disease given their heavy smoking burden.

## Results

### Study cohort

Of the 24,770 study participants, 22,382 (91.4%) were Caucasian, 14,653 (59.2%) were men and 12,886 (52.0%) were current smokers. Participants with a maximum aortic diameter ≥4.5 cm were more likely men (77.9% vs. 58.8% in the those with a maximum aortic diameter <4.5 cm), were heavier smokers, and had a higher prevalence of hypertension and diabetes (all *p* < 0.001). Detailed characteristics are provided in Table 1.

**Table 1:** Participant characteristics

	Entire Cohort	Diameter		Volume		Calcifications		
		<4.5 cm	≥4.5 cm	<210 ml	≥210 ml	1st tertile	2nd tertile	3rd tertile
*N*	24,770	24,490	280	12,385	12,385	8257	8257	8256
Age (years)	61.4 ± 5.0	61.4 ± 5.0	64.1 ± 5.3	60.2 ± 4.5	62.6 ± 5.2	59.2 ± 3.9	61.0 ± 4.6	64.1 ± 5.2
Sex (female)	40.8% (10,117)	41.2% (10,083)	12.1%(34)	66.1% (8188)	15.6% (1929)	46.8% (3867)	39.6% (3270)	36.1% (2980)
BMI (kg/m^2^)	27.9 ± 5.0	27.9 ± 5.0	29.4 ± 4.9	27.5 ± 5.3	28.3 ± 4.7	27.0 ± 4.3	28.1 ± 5.0	28.6 ± 5.6
Race								
White	91.4% (22,643)	91.4% (22,382)	93.2% (261)	90.9% (11,256)	91.9% (11,387)	91.8% (7578)	90.8% (7502)	91.6% (7563)
African American	4.2% (1049)	4.2% (1040)	3.2%(9)	4.3% (533)	4.2% (516)	5.1% (418)	4.4% (367)	3.2% (264)
Others	4.4% (1078)	4.4% (1068)	3.6%(10)	4.8% (596)	3.9% (482)	3.2% (261)	4.7% (388)	5.2% (429)
Smoking Status								
Former	52.0% (12,886)	51.9% (12,723)	58.2% (163)	26.3% (3255)	53.5% (6631)	50.9% (4210)	51.6% (4261)	53.5% (4415)
Current	47.9% (11,884)	48.0% (11,767)	41.7% (117)	49.5% (6130)	46.4% (5754)	49.0% (4047)	48.4% (3996)	46.5% (3841)
Packyears	56.0 ± 24.0	55.9 ± 24.0	61.6 ± 27.4	52.5 ± 21.5	59.5 ± 25.8	51.7 ± 20.9	55.4 ± 23.1	61.4 ± 26.6
Diabetes	9.7% (2392)	9.6% (2355)	13.2%(37)	9.4% (1,165)	9.9% (1227)	5.4% (449)	9.1% (753)	14.4% (1190)
Hypertension	35.1% (8687)	34.9% (8550)	48.9% (137)	30.4% (3771)	39.7% (4916)	23.6% (1948)	33.5% (2.764)	21.9% (812)
History of heart disease	12.9% (3208)	12.8% (3143)	23.2%(65)	9.7% (1207)	16.2% (2001)	6.1% (502)	10.8% (894)	48.1% (3975)
History of stroke	2.8% (690)	2.7% (672)	6.4%(18)	2.3% (289)	3.2% (401)	1.5% (125)	2.3% (190)	4.5% (375)
Aortic features								
Max. Diameter (cm)	3.6 ± 0.4	3.6 ± 0.3	5.0 ± 0.3	3.3 ± 0.2	3.8 ± 0.3	3.5 ± 0.4	3.6 ± 0.4	3.7 ± 0.4
Aortic Volume (ml)	215 ± 52	214 ± 50	342 ± 63	174 ± 21	256 ± 38	200 ± 47	213 ± 50	231 ± 54
Calcium (mm³)	1326 ± 2208	1311 ± 2179	2695 ± 3753	938 ± 1605	1714 ± 2622	111 ± 81	599 ± 230	3270 ± 2963
CVD mortality	1.8% (440)	1.7% (423)	6.1%(17)	1.1% (140)	2.4% (300)	0.8%(65)	1.1%(94)	3.4% (281)
All-cause mortality	7.0% (1.735)	6.9% (1,697)	13.6%(38)	5.2% (642)	8.8% (1,093)	3.9% (326)	5.7% (471)	11.4% (938)
Follow-up (years) median (IQR)	6.5 [6.1–6.8]	6.5 [6.1–6.8]	6.5 [6.0–6.8]	6.5 [6.1–6.9]	6.5 [6.1–6.8]	6.5 [6.2–6.9]	6.5 [6.1–6.9]	6.4 [6.0–6.8]

*BMI* body mass index, *CVD* cardiovascular disease, *IQR* interquartile ranges.

### Discriminatory capacity of aortic features for incident CV mortality

First, we investigated the discrimination of the different aortic features for incident cardiovascular mortality. Harrel´s c-index for maximum aortic diameter, which is the only currently used measure in clinical routine, was 0.61; 95% CI [0.59-0.62]. For aortic volume and aortic calcifications the c-index was 0.63; 95% CI [0.61–0.65] and 0.70; 95% CI [0.68–0.72], respectively, which were significantly higher compared to the maximum diameter (*p* ≤ 0.02).

Similar results were seen for all-cause mortality (c-index for maximum diameter: 0.58; 95% CI [0.56–0.60] vs. volume: 0.59; 95% CI [0.57–0.61] and calcifications: 0.64; 95% CI [0.62–0.66]; all *p* < 0.001).

### Association between aortic features and mortality

Next, we investigated the association between the different thoracic aortic features and cardiovascular mortality. Over a median follow-up of 6.5 [6.1–6.8] years, 1.8% (440/24,770) suffered cardiovascular death. Those who died were significantly older (61.4 ± 5.0 vs. 63.8 ± 5.5; *p* < 0.001), more likely men (71.6%, 315/440) and more likely to present with high risk aortic features (maximum diameter 3.7 ± 0.4 cm vs. 3.6 ± 0.4 cm; volume 240.7 ± 61.2 ml vs. 214.6 ± 51.8 ml; calcifications 3rd tertile 63.4% (281/440) vs. 32.8% (7975/24330); all *p* < 0.001).

**Fig. 1:**
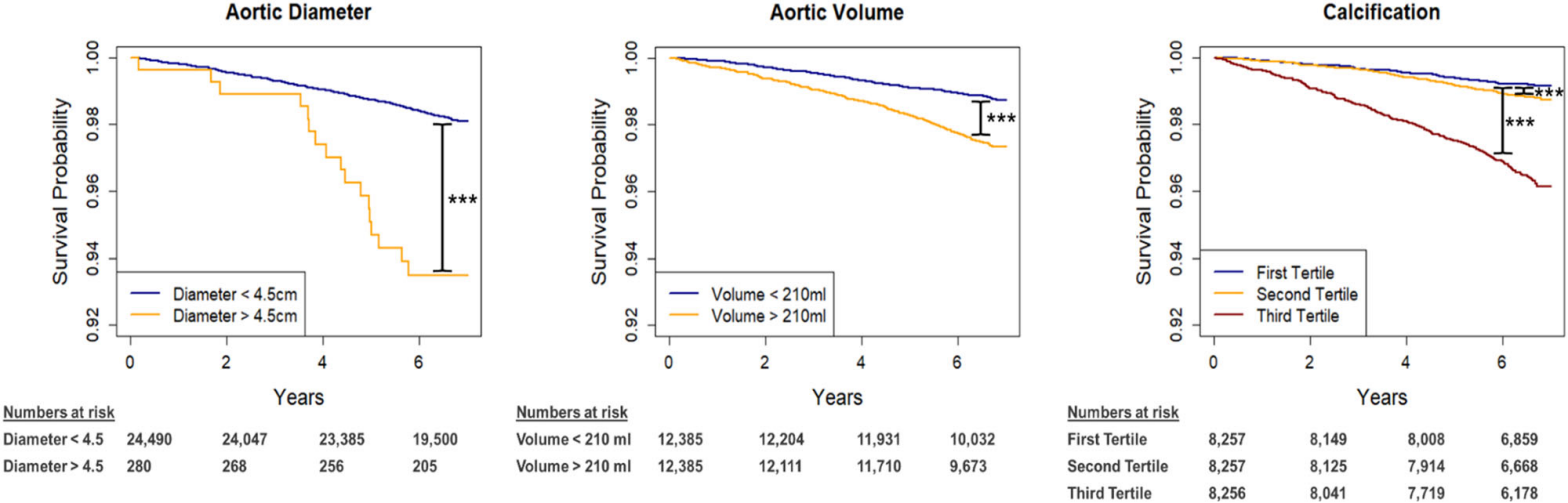
Kaplan Meier survival analysis for the different aortic features to estimate cardiovascular mortality shows worse outcomes for higher risk groups for all evaluated features. Pairwise comparison of survival curves was performed using two-sided Log-Rank tests. *P* values are adjusted for multiple comparisons using the Bonferroni-Holm method; *p < 0.05; **p < 0.01; ***p < 0.001.

Kaplan-Meier survival estimates showed a significantly worse survival for the high risk groups of all evaluated thoracic aortic features (all *p* ≤ 0.02; Fig. 1). Univariable Cox regression analysis revealed a significant association between all aortic features and cardiovascular mortality. The hazard ratio for diameter ≥4.5 cm and volume ≥210 ml were HR 3.68 [95% CI 2.27–5.97] and HR 2.19 [95% CI 1.79–2.67], respectively. The highest hazard of dying was found for the 3rd calcification tertile (2nd tertile HR 1.46 [95% CI 1.07–2.01]; 3rd tertile HR 4.52 [95% CI 3.45–5.92]; Table 2). This signal remained robust after multivariable adjustment for baseline demographics (age, sex, BMI, smoking status) and cardiovascular risk factors (prevalent diabetes, prevalent hypertension, history of heart disease, history of stroke) for the maximum diameter ≥4.5 cm: aHR 2.16 [95% CI 1.32–3.52], volume ≥210 ml: aHR 1.44 [95% CI 1.13–1.83], and 3rd tertile of calcifications: aHR 2.58 [95% CI 1.92–3.48]; whereas the association for the 2nd tertile of calcifications was attenuated: aHR 1.17 [95% CI 0.84–1.61]; Table 2).

**Table 2:** Univariable and multivariable adjusted hazard ratios for the different aortic features to estimate cardiovascular mortality

Cardiovascular mortality					
Aortic features		No patients (events)	Univariable HR (95% CI)	Multivariable HR^a^ (95% CI)	Multivariable HR^b^ (95% CI)
Diameter	<4.5 cm	24,490 (423)	Reference	Reference	NA
	≥4.5 cm	280 (17)	3.68 (2.27–5.97, *p* < 0.001)	2.16 (1.32–3.52, *p* = 0.002)	NA
Volume	<210 ml	12,385 (140)	Reference	Reference	Reference
	≥210 ml	12,385 (300)	2.19 (1.79–2.67, *p* < 0.001)	1.44 (1.13–1.83, *p* = 0.002)	1.41 (1.13–1.79, *p* = 0.004)
Calcifications	1st tertile	8257 (65)	Reference	Reference	Reference
	2nd tertile	8257 (94)	1.46 (1.07–2.01, *p* = 0.018)	1.17 (0.84–1.61, *p* = 0.35)	1.16 (0.84–1.61, *p* = 0.33)
	3rd tertile	8256 (281)	4.52 (3.45–5.92, *p* < 0.001)	2.58 (1.92–3.48, *p* < 0.001)	2.57 (1.91–3.47, *p* < 0.001)

*HR* hazard ratio, *CI* confidence interval, *BMI* body mass index.

^a^Adjusted for race, age at screening, sex, BMI, smoking status (former vs. current), prevalent diabetes, prevalent hypertension, history of myocardial infarction, and stroke.

^b^Adjusted for the same covariates plus maximum diameter.

Similar results were found in sensitivity analyses using an thoracic aortic diameter threshold of ≥4 cm instead of ≥4.5 cm (Supplementary Fig. 3 and Supplementary Tables 1, 2) and in stratified analyses by sex, age, and in individuals without hypertension or history of cardiovascular disease (defined as no prior stroke or heart disease; Supplementary Figs. 4–7 and Supplementary Tables 3–9). In individuals with prevalent hypertension, only thoracic volume and calcifications were significantly associated with cardiovascular mortality, while the association between aortic diameter and cardiovascular mortality was attenuated (Supplementary Fig. 6 and Supplementary Table 8).

When adding either thoracic aortic volume or calcifications to a baseline model with the above mentioned risk factors and aortic diameter, both aortic volume (aHR 1.41 [95% CI 1.13–1.79]) and the 3rd tertile of aortic calcifications (aHR 2.57 [95% CI 1.91–3.47]) remained independently associated with cardiovascular mortality (Table 2).

To test whether thoracic aortic volume or calcifications carry incremental prognostic value beyond a baseline model including the maximum diameter and the above mentioned risk factors we compared nested Cox proportional hazard models using the likelihood ratio test. Adding either thoracic aortic volume or calcifications to a baseline model with maximum diameter, demographics and cardiovascular risk factors resulted in a modest but significant improvement in predicting cardiovascular mortality compared to the baseline model alone (c-index 0.729; 95% CI [0.709–0.749] vs. c-index 0.734; 95% CI [0.714-0734]; *p* = 0.004 for aortic volume and c-index 0.747; 95% CI [0.727–0.767]; *p* < 0.001 for calcifications).

In a subanalysis limited to participants with available CAC (*n* = 13,898) (demographics see Supplementary Table 10), both thoracic aortic calcifications (aHR 3rd tertile 2.12; 95% CI [1.13–3.24]) and volume (aHR 1.58; 95% CI [1.15–2.18]) remained independently associated with CVD mortality (*p* ≤ 0.004) after adjustment for the abovementioned traditional CVD risk factors and CAC (Table 3). This signal also remained robust in a model including risk factors, CAC and all thoracic aortic features together (Table 3). To investigate the potential clinical relevance of this finding, risk reclassification tables for aortic calcification tertiles vs. CAC categories were calculated. Across all CAC categories, we found increasing cardiovascular mortality rates by aortic calcifications tertiles (Supplementary Table 11).

**Table 3:** Multivariable adjusted hazard ratios for the different aortic features to estimate cardiovascular mortality in 13,898 individuals with available coronary artery calcium score

Cardiovascular mortality					
Aortic features		No patients (events)	Multivariable HR^a^ (95% CI)	Multivariable HR^b^ (95% CI)	Multivariable HR^c^ (95% CI)
Diameter	<4.5 cm	13,741 (251)	Reference	Reference	Reference
	≥4.5 cm	157 (8)	1.66 (0.91–3.78, *p* = 0.16)	1.56 (0.77–3.19, *p* = 0.22)	1.46 (0.72–2.98, *p* = 0.30)
Volume	<210 ml	6988 (74)	Reference	Reference	Reference
	≥210 ml	6910 (185)	1.73 (1.26–2.37, *p* < 0.001)	1.58 (1.15–2.18, *p* = 0.004)	1.51 (1.09–2.07, *p* = 0.01)
Calcifications	1st tertile	4596 (33)	Reference	Reference	Reference
	2nd tertile	4699 (59)	1.40 (0.91–2.15, *p* = 0.13)	1.17 (0.76–1.81, *p* = 0.48)	1.15 (0.75–1.79, *p* = 0.52)
	3rd tertile	4603 (167)	2.95 (1.96–4.44, *p* < 0.001)	2.12 (1.39–3.24, *p* < 0.001)	2.06(1.35–3.15, *p* < 0.001)

*HR* hazard ratio, *CI* confidence interval, *BMI* body mass index.

^a^Adjusted for race, age, sex, BMI, smoking status (former vs. current), prevalent diabetes, prevalent hypertension, history of myocardial infarction, and stroke.

^b^Adjusted for the same covariates plus coronary artery calcifications.

^c^Adjusted for the same covariates plus coronary artery calcifications and all aortic features simultaneously.

Adding either thoracic aortic volume or calcifications to a baseline model with risk factors and CAC, both aortic volume (c-index 0.768; 95% CI [0.741–0.795]; *p* = 0.004) and aortic calcificatioins (c-index 0.772; 95% CI [0.747–0797]; *p* < 0.001) resulted in a minor but significant improvement in predicting CVD mortality compared to the model without aortic calcification or volume (c-index 0.764; 95% CI [0.737*–*0.791]).

In a sensitivity analysis, a largely similar pattern as for cardiovascular mortality was found for all-cause mortality in the entire cohort and in stratified analysis by sex, age, hypertension, and in individuals without history of cardiovascular disease (Supplementary Tables 1–10, 12, 13 and Supplementary Figs. 8–12).

## Discussion

In this study, we introduce a fully automated deep learning framework to quantify features of thoracic aortic disease on non-contrast chest CT and identify individuals at increased risk for cardiovascular mortality beyond traditional risk factors. Opportunistic quantification of this currently unused imaging information could complement established risk assessment strategies (e.g., ASCVD risk score) to identify individuals who may benefit from personalized prevention. Our major findings are that 1) both thoracic aortic volume and aortic calcifications allow for a significantly better discrimination of cardiovascular mortality than the maximum thoracic aortic diameter, that 2) thoracic aortic volume, and aortic calcifications carry independent and incremental prognostic value for cardiovascular mortality beyond traditional clinical risk factors and CAC and that 3) in individuals without prevalent hypertension only thoracic aortic volume and calcifications but not thoracic aortic diameter allow for identifying individuals at increased risk of cardiovascular mortality.

These results are of clinical importance as the only currently used parameter for risk assessment and surgical intervention is the maximum aortic diameter. More complex features like alterations of aortic wall structures, elongation, dilation or calcifications are not routinely quantified as automatic and reliable analysis tools are not broadly available. With recent advances in artificial intelligence, several studies have demonstrated the potential of deep learning to accurately assess these features in an automated fashion and have shown their correlation with traditional cardiovascular risk factors^20,21^. However, information on the prognostic value for cardiovascular risk assessment in large scale and high risk populations is limited^22^. Our results in this study demonstrate that thoracic aortic volume and calcifications, which can be quantified from almost any chest CT, have better discrimination and incremental value to predict cardiovascular mortality beyond traditional risk factors and the maximum diameter. Moreover, in a subanalysis of individuals with available coronary artery calcium score, which is the most widely used imaging test to estimate cardiovascular risk^23^, we found that both thoracic aortic calcifications and volume carry incremental prognostic value to predict cardiovascular mortality compared to traditional risk factors and coronary artery calcium alone.

In contrast to CAC, which are a proxy for atherosclerotic changes at the intima layer of the medium size vessel bed, aortic calcifications predominantly occur in the media and reflect the atherosclerotic burden of large vessels, which are known to have an earlier onset and a higher prevalence than CAC^24,25^. Opportunistic quantification of thoracic aortic calcifications may therefore serve as an early warning sign that could complement or even forego coronary artery calcium scanning to identify individuals with an increased cardiovascular risk before CAC become apparent^26^. This highlights a potential clinical use case of our approach as deep learning-based segmentation algorithms and the proposed processing pipelines are publicly available. However, we do not argue to acquire chest CTs for cardiovascular risk assessment as a screening test. Instead, integrating the proposed model into the electronic medical record system as an automated tool to analyze existing non-contrast chest CTs in an opportunistic fashion could improve cardiovascular risk assessment with minimal disruption of current clinical workflows and low additional cost. In this context, the proposed framework could be used as a warning system to identify individuals eligible for established screening, prevention, and surveillance programs.

State-of-the-art imaging of the aorta is performed using contrast agents and ECG-gating to allow for accurate assessment of the vessel wall and account for pulsation artifacts^27^. In contrast, the proposed framework was developed using low-dose non contrast-enhanced, non-gated chest CT scans to investigate their potential value for opportunistic cardiovascular risk assessment in a high risk population of individuals participating in a lung cancer screening trial. Based on visual inspection of a random subset of cases, this is the most likely explanation for a slightly lower Dice coefficient compared to other published models using contrast-enhanced and/or ECG-gated scans. Nevertheless, our results suggest that the proposed framework allows for a reliable assessment of thoracic aortic features even without ECG-gating and contrast agent administration. We consider this as a particular strength of our study considering the already established and upcoming lung cancer screening programs in several countries where our proposed framework could opportunistically quantify prognostically relevant information in high-risk populations for cardiovascular disease that currently goes unnoticed. As the developed framework does not require human input, it provides an ‘end-to-end’ solution for accurate and time-efficient cardiovascular risk assessment for population-wide opportunistic and standardized screening programs with the potential to guide patient management and advance precision medicine.

There are limitations that need to be considered. First, although NLST was a multicenter trial, model performance was not externally validated to test for generalizability in other populations including patients scanned on latest generation CT scanners and with contrast agent. Second, most participants were non-Hispanic white, and dedicated subgroup analysis by race was not possible given the small sample size. Third, the current analysis only included heavy smokers participating in a lung screening trial. Whether our findings translate to other clinical settings needs to be further investigated, i.e., as current lung cancer screening guidelines in the USA now include lower risk populations with a smoking history of 20 pack years. Fourth, no lipid panel was available to adjust for the ASCVD risk score. Lastly, in this exploratory analysis we investigated and extracted different aortic features focusing on the entire thoracic aorta and not considering individual segments (e.g., ascending aorta vs. aortic arch). Whether these individual segments could further refine prognostication needs to be investigated in future studies.

In conclusion, deep learning can quantify features of thoracic aortic disease on non-contrast chest CT which can be used to predict cardiovascular mortality in heavy smokers beyond maximum aortic diameter, traditional cardiovascular risk factors, and CAC. These findings may enable opportunistic risk assessment to improve personalized prevention and treatment strategies and provide the rationale for future prospective trials aiming to evaluate the full clinical potential and impact on decision-making and patient outcomes.

## Methods

### Data source

In this retrospective study, we developed and tested a fully automated deep learning framework to segment the thoracic aorta on non-contrast lung screening chest CT scans to quantify features of thoracic aortic disease and investigated their association with cardiovascular and all-cause mortality in the NLST^28^.

NLST was a randomized controlled trial comparing chest radiographs (CXR) vs. low-dose CT imaging for lung cancer screening. The trial included a community-based cohort of asymptomatic heavy smokers (≥30 pack-years) aged 55–74 years, who were recruited at 21 US sites between August 2002 and April 2004. All participants received a baseline CT (T0) after enrollment and up to two annual follow-up scans (T1 and T2) if no cancer was detected. Relevant exclusion criteria were history of lung cancer or any cancer treatment (except for nonmelanoma skin cancer or carcinoma in situ) within the preceding 5 years. The primary outcome of the trial was a 20% reduction in lung cancer mortality over 6 years with chest CT (*n* = 26,722) compared to CXR^28^.

**Fig. 2:**
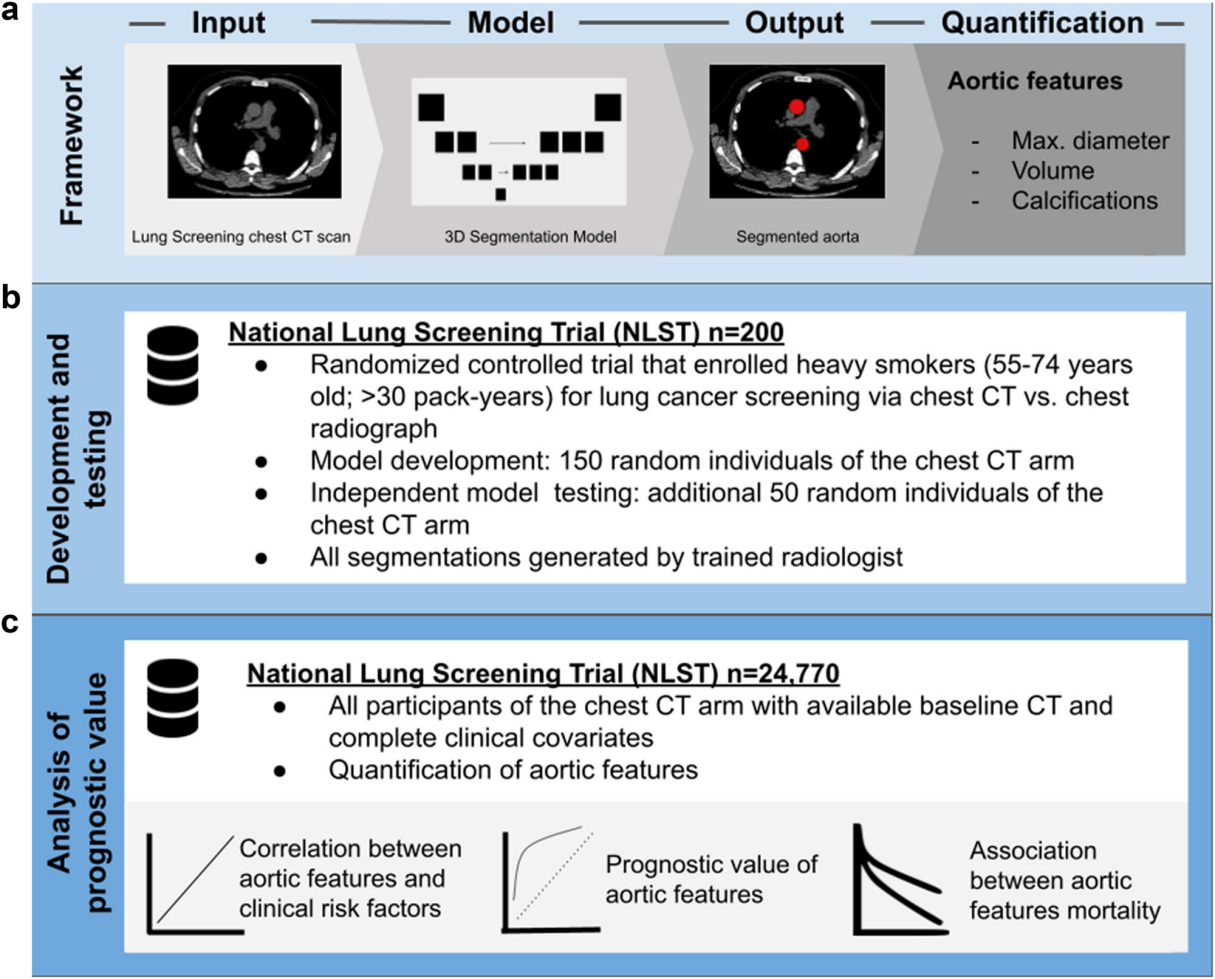
Overview of the study design. **a** The framework was developed to automatically quantify features of aortic disease defined as 1) maximum diameter, 2) volume and 3) calcifications on non-contrast-enhanced lung screening chest CT. **b** For development, a random sample of 150 NLST participants was used. Independent testing was performed in an additional random sample of 50 participants not seen during development. **c** The prognostic value of the aortic features was investigated in 24,770 NLST participants. CT computed tomography, NLST National lung screening trial.

For the current study, only participants from the CT arm with an available T0 CT scan were included. CTs scans were downloaded in DICOM format and soft kernel reconstructions were preferred over hard kernels whenever available. For further analysis, all CT scans were resampled to a resolution of 1.5 × 1.5 × 3 mm to account for different acquisition and reconstruction parameters from multiple sites and different scanners. Participants were excluded if 1) the T0 CT scan was missing or corrupted (*n* = 808), if 2) one or more clinical risk factors (details are provided in the section “Risk factors”) were missing (*n* = 254) or if 3) one or more of the extracted aortic features showed implausible results (*n* = 890), which were a priori defined as a diameter <2.0 cm or a volume <70 ml, which resulted in a final study cohort of 24,770 individuals. An overview of the study design is provided in Fig. 2 and as Consort diagram in Supplementary Fig. 1.

NLST participants initially provided written informed consent for the original trial. Secondary use was approved by the National Cancer Institute in Bethesda, Maryland, the American College of Radiology Imaging Network, and our institutional review board. Our study complies with the declaration of Helsinki and later amendments.

### Model development and testing

#### Development

We developed a fully automated deep learning framework^29^ to segment the thoracic aorta on non-contrast lung screening chest CTs and quantify features of aortic disease. For model development, a random sample of 150 participants was used. Manual segmentations of the thoracic aorta were generated by a trained radiologist on axial reformatted CT scans blinded to demographic features. Additional proofreading and correction, if necessary, was performed by a board-certified radiologist. All segmentations were generated on a local instance of the publicly available post processing platform NORA (www.nora-imaging.com).

The model was trained using the recently introduced deep neuronal patchwork segmentation architecture based on hierarchical and nested stacking of patch-based 3D networks of fixed matrix size but decreasing physical input size, which allows for addressing the dilemma between global context and memory limitation in high-resolution 3D data^29^. The size of the five-layer hierarchical pyramid was chosen to allow for a reasonable 3D field-of-view of each dimension of 80% of the whole matrix size in the coarsest layer and a high spatial resolution in the smallest layer with 1.5 × 1.5 × 3 mm. The matrix size of 32^3^ voxels was selected in a way that would map representative portions of the anatomy. The architecture of the basis U-Net employed was close to the default U-Net configuration with feature dimensions (8, 16, 32, 64) and maximum pooling in the encoding layers and transposed convolutions in the decoding layers. The network was trained for 5 million patches with the Adam optimizer and a learning rate of 0.001. As a loss function, a binary cross-entropy variant of the top-K loss was used^30^. The training took around 20 h with a batch size of 200 images in the graphic unit's memory. Training was performed on a GPU-accelerated server system using an RTX A6000 graphics processing unit (NVIDIA, Santa Clara, CA, USA). During training, patches were randomly sampled so that approximately 80% of the finest patches contained at least one label. No systematic tuning was done with the settings adapted to prior established values^31^.

Individual aortic features were quantified as follows: first, the centerline of the segmentation mask was determined, which was defined as a line along the segmentation mask with equidistant distance from the edges in an orthogonal level at any given point. The maximum diameter was calculated as the maximum distance between the edges of the segmentation mask orthogonally to the centerline. Volume was calculated as voxel volume by summing all voxels within the segmentation mask. Aortic calcifications were defined as voxels with HU values over 130 within the segmentation mask after smoothing the data using a full-width at half maximum (FWHM).*Testing:* Independent testing was performed on an additional 50 randomly selected participants not seen during any part of the model development, who were labeled similarly to the training set. Model performance was assessed by comparing the automatically generated aorta segmentations to the manual segmentations using the Dice coefficient, 95% Hausdorff distance, and Pearson’s correlation coefficient. Compared to manual expert annotations, the model demonstrated a high performance in an independent testing dataset (*n* = 50) not seen during any part of model development with a Dice coefficient of 89.5 ± 0.03, a 95% and Hausdorff distance of 19.1 ± 12.9 for the segmentation mask. For the individual aortic features Pearson’s correlation coefficients were *r* = 0.93 (*p* < 0.001) for maximum diameter, *r* = 0.97 (*p* < 0.001) for volume, and *r* = 0.99 (*p* < 0.001) for calcifications, respectively.

For further quality control, manual assessment of the segmentation masks was performed in a random subset of 1% (*n* = 250) of the participants. In this sample, no systematic failures of the deep learning framework were observed. An image example is presented in Supplementary Fig. 2.

### Aortic features

The final model was locked and applied to all 24,770 included NLST participants with clinical data available to segment the thoracic aorta and quantify features of thoracic aortic disease. The following features were extracted: (1) Maximum diameter (cm) of the thoracic aorta perpendicular to the aortic centerline, (2) total aortic volume (ml) by calculating the voxel volume of the segmentation mask, which in contrast to a 2D diameter measurement at a single location accounts for a general dilated arteriopathy along the entire vessel. (3) thoracic aortic wall calcifications (mm³), defined as all voxels with Hounsfield units ≥130 as a measure of atherosclerosis^32^. To account for potential biases caused by image noise, the data was smoothed using a full-width at half maximum of 3 mm before quantification^33^.

### Risk factors

The following a priori defined clinical covariates with probable prognostic value were included: race (African American, White, others), age at screening, sex, body mass index (BMI; calculated as weight in kg/height in m^2^), smoking status (former vs. current), prevalent diabetes, prevalent hypertension, history of myocardial infarction, and stroke. All covariates were self-reported. In a subanalysis, we also included CAC, which were recently published for 14,959 NLST participants^10^.

### Outcomes

The primary endpoint was death due to cardiovascular disease based on International Classification of Diseases (ICD) 9 codes. The primary endpoint was death due to cardiovascular disease based on International ICD 9 codes^34^: I10, I38, I48, I64, I110, I119, I120, I132, I210, I211, I219, I248, I249, I250, I251, I255, I259, I269, I272, I279, I288, I348, I350, I359, I420, I422, I429, I442, I449, I461, I469, I490, I499, I500, I509, I516, I519, I609, I619, I629, I630, I633, I639, I652, I671, I679, I709, I710, I711, I712, I713, I714, I719, I720, I739, I740, I770.

The secondary endpoint was all-cause mortality. Participants were followed up until the end of 2009, or for a duration of up to 8 years. Mortality was assessed through annual questionnaires, communication with next of kin, and cross-referencing with the National Death Index.

### Statistical analysis

Data are presented as mean ± standard deviation or median and interquartile ranges for continuous variables and as absolute frequencies and percentages for categorical variables. Group differences were assessed by the Chi-squared test and Mann–Whitney *U*-test.

To measure the discriminatory capacity of the different thoracic aortic features for cardiovascular and all-cause mortality, we calculated and compared Harrel´s c-statistic using the R-package “compareC”.

Next, the continuous measures were categorized into groups: For the maximum diameter, the clinically established threshold of ≥4.5 cm defining an ascending aortic aneurysm^27^ as well as ≥4 cm defining aortic dilatation were used^27^. Thoracic aortic volume was dichotomized using a median split (<210 ml vs. ≥210 ml). Thoracic aortic calcifications were categorized into tertiles using the following mm³ cutoffs: low <270, intermediate ≥270–1050, high ≥ 1050. CAC measures were stratified using established Agatston categories^10,35^.

To estimate the time to cardiovascular and all-cause mortality, Kaplan-Meier survival curves and log-rank tests were computed. The association between the different thoracic aortic features and cardiovascular as well as all-cause mortality as assessed using univariable and multivariable Cox proportional hazards regression analyses. Multivariable models were adjusted for the following covariates: age, sex, race, smoking status, BMI, prevalent diabetes mellitus, prevalent hypertension, history of stroke, and history of myocardial infarction and CAC if available. To test whether thoracic aortic features carry incremental prognostic value beyond a baseline model including the maximum diameter and the above-mentioned clinical risk factors we compared nested Cox proportional hazard models using the likelihood ratio test.

In addition, sensitivity analyses stratified by (i) sex, (ii) age (<65 vs. ≥65-years-old), (iii) hypertension, and (iv) individuals without history cardiovascular disease, defined as no prior stroke or heart disease were performed. All *p* values are two-sided and considered to indicate statistical significance if <0.05. All statistical analyses were performed using R (version 4.2.1, https://www.R-project.org/).

### Clinical perspectives

#### What is new?

- Deep learning can automatically quantify imaging features of aortic disease on chest CTs which in turn allows for identification of individuals at high risk for cardiovascular mortality.
- Aortic calcifications and volume provide better discrimination for cardiovascular mortality than maximum aortic diameter, the only currently used measure in clinical routine.
- Aortic calcifications and volume carry independent and incremental prognostic value to estimate cardiovascular mortality beyond traditional clinical risk factors and CAC.

#### Clinical implications

- Aortic volume and calcifications could complement current guideline recommend approaches for risk assessment to identify individuals at increased risk for cardiovascular mortality.
- Deep learning-based opportunistic screening using existing routine chest CTs may improve personalized prevention and risk stratification in clinical care and organized screening programs.

## Supplementary information

Supplementary Information

## Data Availability

The original NLST data can be downloaded upon request from the National Cancer Institute (NLST: https://biometry.nci.nih.gov/cdas/nlst/).

## Code availability

All the code of the deep learning framework is available on bitbucket.org/reisert/patchwork.
