# Supplementary material for: Deep learning to predict cardiovascular mortality from aortic disease in heavy smokers
Source: NPJ Cardiovasc Health. 2024 Nov 6;1:28. doi: 10.1038/s44325-024-00029-3 (PMC12912419; doi:10.1038/s44325-024-00029-3)
Supplement: Supplementary file 1 — Supplementary Information [file 44325_2024_29_MOESM1_ESM.pdf]

Deep learning to predict cardiovascular mortality from aortic angiopathy in heavy smokers

Alexander Rau, MD; Lea Michel, MD; Ben Wilhelm, MSc; Vineet K. Raghu, PhD; Marco Reisert, PhD; Matthias Jung, MD; Elias Kellner, PhD; Christopher L. Schlett, MD, MPH; Hugo J.W.L. Aerts, PhD; Michael T. Lu, MD, MPH; Fabian Bamberg, MD, MPH; Jakob Weiss, MD

I Supplementary Tables

- **Supplementary Table 1** Subgroup characteristics
- **Supplementary Table 2** Univariable and multivariable adjusted hazard ratios for different aortic diameter cutoffs to estimate cardiovascular and all-cause mortality
- **Supplementary Table 3** Univariable and multivariable adjusted hazard ratios for the different aortic features in male participants to estimate cardiovascular and all-cause mortality
- **Supplementary Table 4** Univariable and multivariable adjusted hazard ratios for the different aortic features in female participants to estimate cardiovascular and all-cause mortality
- **Supplementary Table 5** Univariable and multivariable adjusted hazard ratios for the different aortic features in participants younger than 65 years to estimate cardiovascular and all-cause mortality
- **Supplementary Table 6** Univariable and multivariable adjusted hazard ratios for the different aortic features in participants older than 65 years to estimate cardiovascular and all-cause mortality
- **Supplementary Table 7** Univariable and multivariable adjusted hazard ratios for the different aortic features in participants without hypertension to estimate cardiovascular and all-cause mortality
- **Supplementary Table 8** Univariable and multivariable adjusted hazard ratios for the different aortic features in participants with hypertension to estimate cardiovascular and all-cause mortality
- **Supplementary Table 9** Univariable and multivariable adjusted hazard ratios for the different aortic features in participants without history of cardiovascular disease to estimate cardiovascular and all-cause mortality
- **Supplementary Table 10** Characteristics of participants with available coronary artery calcium score
- **Supplementary Table 11** Reclassification table for cardiovascular mortality by aortic calcification tertiles vs. coronary artery calcium score categories
- **Supplementary Table 12** Univariable and multivariable adjusted hazard ratios for the different aortic features to estimate all-cause mortality
- **Supplementary Table 13** Univariable and multivariable adjusted hazard ratios for the different aortic features and coronary artery calcification to estimate all-cause mortality

II Supplementary Figures

- **Supplementary Figure 1** Consort diagram
- **Supplementary Figure 2** Performance of the deep learning framework

- **Supplementary Figure 3** Kaplan Meier survival analysis for the different aortic diameter cutoffs to estimate cardiovascular and all-cause mortality
- **Supplementary Figure 4** Kaplan Meier survival analysis for the different aortic features to estimate cardiovascular mortality stratified by sex
- **Supplementary Figure 5** Kaplan Meier survival analysis for the different aortic features to estimate cardiovascular mortality stratified by age
- **Supplementary Figure 6** Kaplan Meier survival analysis for the different aortic features to estimate cardiovascular mortality stratified by hypertension
- **Supplementary Figure 7** Kaplan Meier survival analysis for the different aortic features to estimate cardiovascular mortality in individuals without history of cardiovascular disease
- **Supplementary Figure 8** Kaplan Meier survival analysis for the different aortic features to estimate all-cause mortality
- **Supplementary Figure 9** Kaplan Meier survival analysis for the different aortic features to estimate all-cause mortality stratified by sex
- **Supplementary Figure 10** Kaplan Meier survival analysis for the different aortic features to estimate all-cause mortality stratified by age
- **Supplementary Figure 11** Kaplan Meier survival analysis for the different aortic features to estimate all-cause mortality stratified by hypertension
- **Supplementary Figure 12** Kaplan Meier survival analysis for the different aortic features to estimate all-cause mortality in individuals without history of cardiovascular disease

Supplementary Tables

Supplementary Table 1 Subgroup characteristics

	Entire Cohort	Diameter		Sex		Age	
		<4 cm	*≥4 cm*	*Male*	*Female*	*<65 years*	*≥65 years*
**N**	24,770	21,963	2,807	14,653	10,117	18,211	6,559
**Age (years)**	61.4±5.0	61.1±74.9	63.4±5.2	61.6±5.1	61.1±4.9	58.9±2.8	68.3±2.7
**Sex (female)**	40.8% (10,117)	44.4% (9,772)	12.3% (345)	0% (0)	100% (10,117)	41.9% (7,629)	37.9% (2,488)
**BMI (kg/m^2)**	27.9±5.0	27.8±5.0	28.8±4.9	28.2±4.6	27.4±5.6	28.0±5.1	27.5±4.8
**Race**							
White	91.4% (22,643)	91.3% (20,051)	92.3% (2,592)	91.1% (13,345)	91.9% (9,298)	91.1% (16,598)	92.1% (6,045)
African American	4.2% (1,049)	4.3% (940)	3.9% (109)	3.9% (574)	4.7% (475)	4.6% (835)	3.3% (214)
Others	4.4% (1,078)	4.4% (972)	3.8% (106)	5.0% (734)	3.4% (344)	4.3% (778)	4.6% (300)
**Smoking Status**							
Former	52.0% (12,886)	51.6% (11,339)	55.1% (1,547)	53.7% (7,868)	49.6% (5,018)	49.2% (8,952)	59.9% (3,934)
Current	47.9% (11,884)	48.4 (10,624)	44.9% (1,260)	46.3% (6,785)	50.4% (5,099)	50.8% (9,259)	40.0% (2,625)
Packyears	56.0±24.0	55.3±23.4	61.4±27.5	59.4±25.9	51.1±20.0	53.9±22.3	61.7±27.3
**Diabetes**	9.7% (2,392)	9.6% (2,114)	9.9% (278)	11.3% (1,660)	7.2% (732)	9.1% (1,657)	11.2% (735)
**Hypertension**	35.1% (8,687)	33.7% (7,409)	45.5% (1,278)	36.1% (5,297)	33.5% (3,390)	32.5% (5,911)	42.3% (2,776)
**History of heart disease**	12.9% (3,208)	12.2% (2,682)	18.7% (526)	16.7% (2,443)	7.6% (765)	11.0% (2,009)	18.3% (1,199)
**History of stroke**	2.8% (690)	2.7% (591)	3.5% (99)	2.9% (421)	2.7% (269)	2.2% (406)	4.3% (284)
**Aortic features**							
Max. Diameter (cm)	3.6±0.4	3.5±0.3	4.2±0.3	3.7±0.3	3.4±0.3	3.5±0.4	3.7±0.4
Aortic Volume (ml)	215±52	205±43	293±47	239±48	181±37	207±48	238±55
Calcium in mm^3	1,326±2,208	1,234±2,063	2,052±3,018	1,428±2,307	1,179±2,049	888±1,472	2,544±3,221
**CVD mortality**	1.8% (440)	1.6% (349)	3.2% (91)	2.1% (315)	1.2% (125)	1.3% (237)	3.1% (203)
**All-cause mortality**	7.0% (1,735)	6.6% (1,449)	10.1% (286)	8.2% (1,207)	5.2% (528)	5.3% (971)	11.6% (764)
**Follow-up (years)** median (IQR)	6.5 [6.1-6.8]	6.5 [6.1-6.8]	6.5 [6.1-6.8]	6.5 [6.1-6.8]	6.5 [6.1-6.8]	6.5 [6.1-6.8]	6.5 [6.0-6.8]

BMI=body mass index; CVD=cardiovascular disease, IQR=interquartile ranges

Supplementary Table 2 Univariable and multivariable adjusted hazard ratios for different aortic diameter cutoffs to estimate cardiovascular and all-cause mortality

Cardiovascular mortality					
Diameter	**No patients (events)**	**Univariable HR (95% CI)**	**Multivariable aHR* (95% CI)**	**Multivariable aHR** and (95% CI)**	**Multivariable aHR*** and (95% CI)**
< 4.0 cm	21,963 (349)	Reference	Reference	Reference	Reference
≥ 4.0 cm	2,807 (91)	2.08 (1.65-3.62, p<.001)	1.39 (1.11-1.79, p=0.007)	1.29 (1.00-1.65, p=0.04)	1.37 (1.07-1.74, p=0.01)
< 4.5 cm	24,490 (423)	Reference	Reference	Reference	Reference
≥ 4.5cm	280 (17)	3.68 (2.27-5.97, p<.001)	2.16 (1.32-3.52, p=0.002)	2.02 (1.23-3.30, p=0.005)	2.06 (1.26-3.37, p=0.003)
**All-cause mortality**					
	**No patients (events)**	**Univariable HR (95% CI)**	**Multivariable aHR* (95% CI)**	**Multivariable aHR** and Aortic Volume (95% CI)**	**Multivariable aHR*** and Calcifications (95% CI)**
< 4.0 cm	21,963 (1,449)	Reference	Reference	Reference	Reference
≥ 4.0 cm	2,807 (286)	1.58 (1.39-1.79, p<.001)	1.17 (1.02-1.33, p=0.02)	1.11 (0.97-1.27, p=0.12)	1.15 (1.00-1.31, p=0.04)
< 4.5 cm	24,490 (1,697)	Reference	Reference	Reference	Reference
≥ 4.5cm	280 (38)	2.06 (1.49-2.84, p<.001)	1.41 (1.02-1.95, p=0.04)	1.36 (0.98-1.88, p=0.07)	1.37 (0.99-1.89, p=0.06)

*adjusted for adjusted for race, age at screening, sex, BMI, smoking status (former vs. current), prevalent diabetes, prevalent hypertension, history of myocardial infarction, and stroke; **adjusted for the same covariates plus maximum diameter; ***adjusted for the same covariates plus calcifications

HR=hazard ratio; CI=confidence interval, BMI=body mass index

Supplementary Table 3 Univariable and multivariable adjusted hazard ratios for the different aortic features in male participants to estimate cardiovascular and all-cause mortality

Cardiovascular mortality					
		**No patients (events)**	**Univariable HR (95% CI)**	**Multivariable HR* (95% CI)**	**Multivariable HR** (95% CI)**
**Diameter**	< 4.5cm	14,407 (301)	Reference	Reference	NA
	≥ 4.5cm	246 (14)	2.85 (1.67-4.87, p<.001)	1.97 (1.14-3.38, p=0.01)	NA
**Volume**	<210ml	4,197 (62)	Reference	Reference	Reference
	≥210ml	10,456 (253)	1.66 (1.26-2.19, p<.001)	1.31 (0.98-1.75, p=0.07)	1.28 (0.96-1.71, p=0.09)
**Calcifications**	1st tertile	4,390 (48)	Reference	Reference	Reference
	2nd tertile	4,987 (65)	1.21 (0.83-1.75, p=0.33)	1.01 (0.69-1.47, p=0.96)	1.01 (0.69-1.48, p=0.95)
	3rd tertile	5,276 (202)	3.67 (2.68-5.03, p<.001)	2.26 (1.59-3.19, p<.001)	2.25 (1.59-3.19, p<.001)
**All-cause mortality**					
		**No patients (events)**	**Univariable HR (95% CI)**	**Multivariable HR* (95% CI)**	**Multivariable HR** (95% CI)**
**Diameter**	< 4.5cm	14,407 (1,172)	Reference	Reference	NA
	≥ 4.5cm	246 (35)	1.84 (1.31-2.57, p<.001)	1.46 (1.04-2.05, p=0.03)	NA
**Volume**	<210ml	4,197 (258)	Reference	Reference	Reference
	≥210ml	10,456 (949)	1.49 (1.31-1.72, p<.001)	1.23 (1.06-1.42, p=0.005)	1.21 (1.05-1.41, p=0.01)
**Calcifications**	1st tertile	4,390 (226)	Reference	Reference	Reference
	2nd tertile	4,987 (319)	1.26 (1.06-1.49, p=0.008)	1.12 (0.94-1.33, p=0.20)	1.12 (0.94-1.33, p=0.20)
	3rd tertile	5,276 (662)	2.57 (2.21-2.99, p<.001)	1.80 (1.52-2.13, p<.001)	1.79 (1.52-2.13, p<.001)

*adjusted for adjusted for race, age at screening, sex, BMI, smoking status (former vs. current), prevalent diabetes, history of myocardial infarction, and stroke; **adjusted for the same covariates plus maximum diameter

HR=hazard ratio; CI=confidence interval, BMI=body mass index

Supplementary Table 4 Univariable and multivariable adjusted hazard ratios for the different aortic features in female participants to estimate cardiovascular and all-cause mortality

**Cardiovascular mortality**					
		**No patients (events)**	**Univariable HR (95% CI)**	**Multivariable HR* (95% CI)**	**Multivariable HR** (95% CI)**
**Diameter**	< 4.5cm	10,083 (122)	Reference	Reference	NA
	≥ 4.5cm	34 (3)	7.46 (2.37-23.46, p<.001)	4.89 (1.55-15.44, p=0.007)	NA
**Volume**	<210ml	8,188 (78)	Reference	Reference	Reference
	≥210ml	1,929 (47)	2.61 (1.82-3.75, p<.001)	1.70 (1.16-2.50, p=0.007)	1.63 (1.09-2.41, p=0.02)
**Calcifications**	1st tertile	3,867 (17)	Reference	Reference	Reference
	2nd tertile	3,270 (29)	2.04 (1.12-3.71, p=0.02)	1.62 (0.88-2.96, p=0.12)	1.63 (0.89-2.98, p=0.12)
	3rd tertile	2,980 (79)	6.26 (3.71-10.58, p<.001)	3.54 (1.99-6.29, p<.001)	3.53 (1.99-6.28, p<.001)
**All-cause mortality**					
		**No patients (events)**	**Univariable HR (95% CI)**	**Multivariable HR* (95% CI)**	**Multivariable HR** (95% CI)**
**Diameter**	< 4.5cm	10,083 (525)	Reference	Reference	NA
	≥ 4.5cm	34 (3)	1.31 (0.56-5.38, p=0.34)	1.21 (0.29-3.76, p=0.75)	NA
**Volume**	<210ml	8,188 (384)	Reference	Reference	Reference
	≥210ml	1,929 (144)	1.63 (1.35-1.97, p<.001)	1.16 (0.95-1.42, p=0.16)	1.16 (0.94-1.42, p=0.17)
**Calcifications**	1st tertile	3,867 (100)	Reference	Reference	Reference
	2nd tertile	3,270 (152)	1.82 (1.41-2.34, p<.001)	1.57 (1.22-2.03, p=0.001)	1.57 (1.22-2.03, p=0.001)
	3rd tertile	2,980 (276)	3.76 (2.99-4.72, p<.001)	2.50 (1.94-3.23, p<.001)	2.50 (1.94-3.23, p<.001)

*adjusted for adjusted for race, age at screening, sex, BMI, smoking status (former vs. current), prevalent diabetes, history of myocardial infarction, and stroke; **adjusted for the same covariates plus maximum diameter

HR=hazard ratio; CI=confidence interval, BMI=body mass index

Supplementary Table 5 Univariable and multivariable adjusted hazard ratios for the different aortic features in participants younger than 65 years to estimate cardiovascular and all-cause mortality

**Cardiovascular mortality**					
		**No patients (events)**	**Univariable HR (95% CI)**	**Multivariable HR* (95% CI)**	**Multivariable HR** (95% CI)**
**Diameter**	< 4.5cm	18,066 (231)	Reference	Reference	NA
	≥ 4.5cm	145 (5)	3.39 (1.51-7.62, p=0.003)	2.12 (0.94-4.83, p=0.07)	NA
**Volume**	<210ml	10,188 (97)	Reference	Reference	Reference
	≥210ml	8,023 (140)	1.85 (1.43-2.40, p<.001)	1.37 (1.01-1.86, p=0.04)	1.35 (0.99-1.83, p=0.06)
**Calcifications**	1st tertile	7,386 (55)	Reference	Reference	Reference
	2nd tertile	6,424 (79)	1.67 (1.18-2.35, p=0.004)	1.36 (0.96-1.92, p=0.09)	1.36 (0.96-1.93, p=0.08)
	3rd tertile	4,401 (103)	3.24(2.34-4.49, p<.001)	1.98 (1.39-2.85, p<.001)	1.98 (1.39-2.82, p<.001)
**All-cause mortality**					
		**No patients (events)**	**Univariable HR (95% CI)**	**Multivariable HR* (95% CI)**	**Multivariable HR** (95% CI)**
**Diameter**	< 4.5cm	18,066 (957)	Reference	Reference	NA
	≥ 4.5cm	145 (14)	1.92 (1.13-3.26, p=0.02)	1.58 (0.93-2.68, p=0.09)	NA
**Volume**	<210ml	10,188 (454)	Reference	Reference	Reference
	≥210ml	8,023 (517)	1.47 (1.29-1.66, p<.001)	1.16 (1.00-1.35, p=0.01)	1.21 (1.04-1.41, p=0.04)
**Calcifications**	1st tertile	7,386 (266)	Reference	Reference	Reference
	2nd tertile	6,424 (326)	1.43 (1.21-1.68, p<.001)	1.30 (1.11-1.54, p=0.002)	1.30 (1.10-1.54, p=0.001)
	3rd tertile	4,401 (379)	2.49 (2.13-2.91, p<.001)	1.98 (1.67-2.34, p<.001)	1.97 (1.66-2.34, p<.001)

*adjusted for adjusted for race, age at screening, sex, BMI, smoking status (former vs. current), prevalent diabetes, history of myocardial infarction, and stroke; **adjusted for the same covariates plus maximum diameter

HR=hazard ratio; CI=confidence interval, BMI=body mass index

Supplementary Table 6 Univariable and multivariable adjusted hazard ratios for the different aortic features in participants older than 65 years to estimate cardiovascular all-cause mortality

**Cardiovascular mortality**					
		**No patients (events)**	**Univariable HR (95% CI)**	**Multivariable HR* (95% CI)**	**Multivariable HR** (95% CI)**
**Diameter**	< 4.5cm	6,424 (192)	Reference	Reference	NA
	≥ 4.5cm	135 (11)	2.81 (1.53-5.16, p<.001)	2.13 (1.15-3.94, p=0.02)	NA
**Volume**	<210ml	2,197 (43)	Reference	Reference	Reference
	≥210ml	4,362 (160)	1.93 (1.38-2.71, p<.001)	1.58 (1.08-2.33, p=0.02)	1.54 (1.05-2.27, p=0.03)
**Calcifications**	1st tertile	871 (10)	Reference	Reference	Reference
	2nd tertile	1,833 (15)	0.73 (0.33-1.61, p=0.43)	0.67 (0.30-1.50, p=0.33)	0.67 (0.30-1.49, p=0.33)
	3rd tertile	3,855 (178)	4.28 (2.26-8.09, p<.001)	3.45 (1.80-6.56, p<.001)	3.42 (1.79-6.55, p<.001)
**All-cause mortality**					
		**No patients (events)**	**Univariable HR (95% CI)**	**Multivariable HR* (95% CI)**	**Multivariable HR** (95% CI)**
**Diameter**	< 4.5cm	6,424 (740)	Reference	Reference	NA
	≥ 4.5cm	135 (24)	1.60 (1.07-2.40, p=0.02)	1.31 (0.87-1.97, p=0.19)	NA
**Volume**	<210ml	2,197 (188)	Reference	Reference	Reference
	≥210ml	4,362 (576)	1.59 (1.35-1.88, p<.001)	1.28 (1.06-1.55, p=0.01)	1.27 (1.05-1.54, p=0.01)
**Calcifications**	1st tertile	871 (60)	Reference	Reference	Reference
	2nd tertile	1,833 (145)	1.17 (0.87-1.58, p=0.31)	1.11 (0.82-1.49, p=0.51)	1.11 (0.82-1.49, p=0.51)
	3rd tertile	3,855 (559)	2.25 (1.73-2.94, p<.001)	1.89 (1.44-2.49, p<.001)	1.89 (1.44-2.49, p<.001)

*adjusted for adjusted for race, age at screening, sex, BMI, smoking status (former vs. current), prevalent diabetes, history of myocardial infarction, and stroke; **adjusted for the same covariates plus maximum diameter

HR=hazard ratio; CI=confidence interval, BMI=body mass index

Supplementary Table 7 Univariable and multivariable adjusted hazard ratios for the different aortic features in participants without hypertension to estimate cardiovascular and all-cause mortality

Cardiovascular mortality					
		**No patients (events)**	**Univariable HR (95% CI)**	**Multivariable HR* (95% CI)**	**Multivariable HR** (95% CI)**
**Diameter**	< 4.5cm	15,940 (220)	Reference	Reference	NA
	≥ 4.5cm	143 (4)	2.14 (0.79-5.76, p=0.13)	1.39 (0.51-3.76, p=0.52)	NA
**Volume**	<210ml	8,614 (79)	Reference	Reference	Reference
	≥210ml	7,469 (145)	2.16 (1.64-2.84, p<.001)	1.49 (1.07-2.08, p=0.01)	1.49 (1.07-2.07, p=0.02)
**Calcifications**	1st tertile	6,309 (44)	Reference	Reference	Reference
	2nd tertile	5,493 (55)	1.46 (0.98-2.16, p=0.06)	1.17 (0.78-1.75, p=0.44)	1.17 (0.78-1.75, p=0.44)
	3rd tertile	4,281 (125)	4.36 (3.09-6.15, p<.001)	2.59 (1.77-3.79, p<.001)	2.59 (1.77-3.79, p<.001)
**All-cause mortality**					
		**No patients (events)**	**Univariable HR (95% CI)**	**Multivariable HR* (95% CI)**	**Multivariable HR** (95% CI)**
**Diameter**	< 4.5cm	15,940 (994)	Reference	Reference	NA
	≥ 4.5cm	143 (17)	2.03 (1.26-3.28, p=0.003)	1.52 (0.94-2.46, p=0.09)	NA
**Volume**	<210ml	8,614 (405)	Reference	Reference	Reference
	≥210ml	7,469 (606)	1.76 (1.56-2.00, p<.001)	1.22 (1.05-1.42, p=0.01)	1.21 (1.04-1.41, p=0.01)
**Calcifications**	1st tertile	6,309 (220)	Reference	Reference	Reference
	2nd tertile	5,493 (319)	1.55 (1.31-1.83, p<.001)	1.33 (1.11-1.57, p=0.001)	1.33 (1.12-1.58, p=0.001)
	3rd tertile	4,281 (452)	2.91 (2.49-3.41, p<.001)	1.92 (1.61-2.29, p<.001)	1.92 (1.61-2.28, p<.001)

*adjusted for adjusted for race, age at screening, sex, BMI, smoking status (former vs. current), prevalent diabetes, history of myocardial infarction, and stroke; **adjusted for the same covariates plus maximum diameter

HR=hazard ratio; CI=confidence interval, BMI=body mass index

Supplementary Table 8 Univariable and multivariable adjusted hazard ratios for the different aortic features in participants with hypertension to estimate cardiovascular and all-cause mortality

Cardiovascular mortality					
		**No patients (events)**	**Univariable HR (95% CI)**	**Multivariable HR* (95% CI)**	**Multivariable HR** (95% CI)**
**Diameter**	< 4.5cm	8,550 (203)	Reference	Reference	NA
	≥ 4.5cm	137 (21)	4.12 (2.35-7.21, p<.001)	2.62 (1.48-4.63, p<.001)	NA
**Volume**	<210ml	3,771 (61)	Reference	Reference	Reference
	≥210ml	4,916 (155)	2.08 (1.57-2.77, p<.001)	1.34 (0.94-1.89, p=0.10)	1.48 (1.06-2.08, p=0.02)
**Calcifications**	1st tertile	1,948 (21)	Reference	Reference	Reference
	2nd tertile	2,764 (39)	1.32 (0.77-2.24, p=0.31)	1.11 (0.65-1.90, p=0.69)	1.11 (0.65-1.90, p=0.69)
	3rd tertile	3,975 (156)	3.81 (2.41-6.00, p<.001)	2.45 (1.51-3.99, p<.001)	2.45 (1.51-.3.98, p<.001)
**All-cause mortality**					
		**No patients (events)**	**Univariable HR (95% CI)**	**Multivariable HR* (95% CI)**	**Multivariable HR** (95% CI)**
**Diameter**	< 4.5cm	8,550 (703)	Reference	Reference	NA
	≥ 4.5cm	137 (13)	1.93 (1.25-2.98, p=0.003)	1.35 (0.87-2.09, p=0.18)	NA
**Volume**	<210ml	3,771 (237)	Reference	Reference	Reference
	≥210ml	4,916 (487)	1.61 (1.37-1.88, p<.001)	1.19 (0.99-1.43, p=0.05)	1.19 (0.99-1.43, p=0.07)
**Calcifications**	1st tertile	1,948 (86)	Reference	Reference	Reference
	2nd tertile	2,764 (152)	1.25 (0.96-1.63, p=0.09)	1.09 (0.84-1.43, p=0.50)	1.09 (0.84-1.43, p=0.50)
	3rd tertile	3,975 (486)	2.91 (2.32-3.67, p<.001)	2.04 (1.59-2.61, p<.001)	2.04 (1.59-2.61, p<.001)

*adjusted for adjusted for race, age at screening, sex, BMI, smoking status (former vs. current), prevalent diabetes, history of myocardial infarction, and stroke; **adjusted for the same covariates plus maximum diameter

HR=hazard ratio; CI=confidence interval, BMI=body mass index

Supplementary Table 9 Univariable and multivariable adjusted hazard ratios for the different aortic features in participants without history of cardiovascular disease to estimate cardiovascular and all-cause mortality

**Cardiovascular mortality**					
		**No patients (events)**	**Univariable HR (95% CI)**	**Multivariable HR* (95% CI)**	**Multivariable HR** (95% CI)**
**Diameter**	< 4.5cm	20,862 (282)	Reference	Reference	NA
	≥ 4.5cm	205 (11)	4.14 (2.26-7.56, p<.001)	2.71 (1.47-4.97, p=0.001)	NA
**Volume**	<210ml	10,956 (95)	Reference	Reference	Reference
	≥210ml	10,111 (198)	2.30 (1.80-2.94, p<.001)	1.63 (1.21-2.19, p=0.001)	1.63 (1.21-2.19, p=0.001)
**Calcifications**	1st tertile	7,649 (54)	Reference	Reference	Reference
	2nd tertile	7,213 (71)	1.41 (0.99-2.01, p=0.06)	1.15 (0.81-1.66, p=0.43)	1.16 (0.81-1.66, p=0.43)
	3rd tertile	6,205 (168)	3.98 (2.93-5.41, p<.001)	2.49 (1.78-3.49, p<.001)	2.49 (1.78-3.49, p<.001)
**All-cause mortality**					
		**No patients (events)**	**Univariable HR (95% CI)**	**Multivariable HR* (95% CI)**	**Multivariable HR** (95% CI)**
**Diameter**	< 4.5cm	20,862 (1,276)	Reference	Reference	NA
	≥ 4.5cm	205 (26)	2.18 (1.48-3.21, p<.001)	1.56 (1.06-2.31, p=0.03)	NA
**Volume**	<210ml	10,956 (491)	Reference	Reference	Reference
	≥210ml	10,111 (811)	1.83 (1.64-2.05, p<.001)	1.27 (1.11-1.46, p<.001)	1.26 (1.10-1.49, <.001)
**Calcifications**	1st tertile	7,649 (287)	Reference	Reference	Reference
	2nd tertile	7,213 (377)	1.41 (1.21-1.65, p<.001)	1.23 (1.05-1.44, p=0.001)	1.23 (1.05-1.44, p=0.01)
	3rd tertile	6,205 (638)	2.87 (2.49-3.30, p<.001)	2.02 (1.73-2.36, p<.001)	2.02 (1.73-2.36, p<.001)

*adjusted for adjusted for race, age at screening, sex, BMI, smoking status (former vs. current), prevalent diabetes, prevalent hypertension; **adjusted for the same covariates plus maximum diameter

HR=hazard ratio; CI=confidence interval, BMI=body mass index

Supplementary Table 10 Characteristics of participants with available coronary artery calcium score

	Entire Cohort	Diameter		Volume		Aortic Calcifications			Coronary Artery Calcifications			
		<4.5 cm	*≥4.5 cm*	*<210 ml*	*≥210 ml*	*1st tertile*	*2nd tertile*	*3rd tertile*	*Category 0*	*Category 1*	*Category 2*	*Category 3*
**N**	13,898	13,741	157	6,988	6,910	4,596	4,699	4,603	3,361	4,407	2,193	3,937
**Age (years)**	61.4±5.0	61.4±5.0	64.2±5.3	60.2±4.5	62.7±5.2	59.2±3.9	61.0±4.6	64.1±5.2	59.8±4.3	60.7±4.7	61.9±5.1	63.4±5.3
**Sex (female)**	41.2% (5,728)	41.5% (5,709)	12.1% (19)	66.4% (4,639)	15.8% (1,089)	47.3% (2,178)	39.9% (1,878)	36.3% (1,672)	58.3% (1,960)	47.6% (2,098)	35.2% (771)	22.8% (899)
**BMI (kg/m²)**	27.9±5.1	27.9±5.0	29.6±4.9	27.4±5.3	28.3±4.8	26.9±4.4	28.1±5.1	28.6± 5.6	27.4± 5.0	27.8± 5.1	28.1± 5.2	28.2± 5.3
**Race**												
White	91.1% (12,658)	91.0% (12,511)	93.6% (147)	90.7% (6,339)	91.4% (6,319)	91.4% (4,201)	90.9% (4,270)	90.9% (4,187)	90.1% (3,030)	90.9% (4,004)	90.7% (41,988)	92.4% (3,636)
African American	4.4% (616)	4.4% (611)	3.2% (5)	4.5% (316)	4.3% (300)	5.5% (252)	4.3% (202)	3.5% (162)	5.4% (183)	5.1% (225)	4.4% (96)	2.8% (112)
Others	4.5% (624)	4.5% (619)	3.2% (5)	4.8% (333)	4.2% (290)	3.1% (143)	4.8% (227)	5.5% (254)	4.4% (148)	4.0% (178)	4.9% (109)	4.8% (189)
**Smoking Status**												
Former	51.8% (7,202)	51.7% (7,110)	58.6% (92)	50.0% (3,500)	53.6% (3,702)	50.9% (2,342)	51.6% (2,423)	52.9% (2,437)	49.9% (1,678)	50.9% (2,242)	51.5% (1,130)	54.7% (2,152)
Current	48.2% (6,696)	48.3% (6,631)	41.4% (65)	50.0% (3,488)	46.4% (3,208)	49.0% (2,254)	48.4% (2,276)	47.1% (2,166)	50.0% (1,683)	49.1% (2,165)	48.5% (1,063)	45.3% (1,785)
Packyears	55.9±23.8	55.9±23.7	62.5±28.9	52.5±21.1	59.4±25.8	51.2±20.6	55.3±23.1	61.4±26.3	51.5±20.5	54.3±23.0	57.3±25.2	60.9±25.6
**Diabetes**	9.5% (1,315)	9.4% (1,297)	11.5% (18)	9.1% (635)	9.8% (680)	5.2% (239)	8.9% (414)	14.4% (662)	6.1% (204)	7.1% (313)	10.1% (221)	14.1% (557)
**Hypertension**	35.4% (4,924)	35.3% (4,854)	44.6% (70)	30.6% (2,139)	40.3% (2,785)	24.1% (1,108)	33.6% (1,579)	48.6% (2,237)	26.6% (893)	31.8% (1,403)	37.8% (829)	45.7% (1,799)
**History of heart disease**	12.9% (1,794)	12.8% (1,754)	25.5% (40)	9.6% (672)	16.2% (1,122)	6.3% (289)	10.6% (497)	21.9% (1,008)	4.6% (153)	5.9% (153)	11.2% (245)	28.8% (1,135)
**History of stroke**	2.7% (380)	2.7% (373)	4.5% (7)	2.3% (161)	3.2% (219)	1.4% (64)	2.4% (112)	4.4% (204)	1.9% (63)	2.1% (92)	3.1% (67)	4.0% (158)
**CAC score**	356±710	352±706	718±984	216±529	499±832	105±268	259±503	706±1000	0±0	31±28	185±57	1121±976
**Aortic features**												
Max. Diameter (cm)	3.6±0.4	3.6±0.3	4.8±0.4	3.3±0.2	3.8±0.3	3.5±0.3	3.6±0.4	3.7±0.4	3.5±0.3	3.5±0.4	3.6±0.3	3.7±0.4
Aortic Volume (ml)	215±52	214±50	344±67	174±24	257±39	200±47	213±50	232±55	194±47	209±50	221±49	236±55

Calcium (mm³)	1,300±2,157	1,288±2,139	2,346±3,199	921±1,555	1,682±2,574	111±81	595±229	3,207 ±2,904	514 ±980	820 ±1,223	1,340 ±1,777	2,486 ±3,160
**CVD mortality**	1.9% (259)	1.8% (251)	5.1% (8)	1.1% (74)	2.7% (185)	0.7% (33)	1.3% (59)	3.6% (167)	0.5% (18)	1.1% (47)	2.3% (51)	3.6% (143)
**All-cause mortality**	7.2% (995)	7.1% (975)	12.7% (20)	5.4% (377)	8.9% (618)	3.7% (172)	6.0% (284)	11.7% (539)	4.1% (139)	5.2% (228)	7.6% (167)	11.7% (461)
**Follow-up (years)** median (IQR)	6.5 [6.1-6.8]	6.5 [6.1-6.8]	6.5 [6.0-6.8]	6.5 [6.1-6.9]	6.5 [6.1-6.8]	6.5 [6.2-6.9]	6.5 [6.1-6.9]	6.4 [6.0-6.8]	6.5 [6.1-6.9]	6.5 [6.1-6.8]	6.5 [6.1-6.9]	6.5 [6.0-6.8]

BMI=body mass index; CAC=coronary artery calcifications; CVD=cardiovascular disease, IQR=interquartile ranges

Supplementary Table 11 Reclassification table for cardiovascular mortality by aortic calcification tertiles vs. coronary artery calcium score categories

		**Coronary artery calcium score**			
		**0**	**1**	**2**	**3**
**Aortic Calcifications**	**1st tertile**	0.36% (7/1,397)	0.85% (14/1,639)	0.74% (4/537)	1.66% (8/483)
	**2nd tertile**	0.50% (5/1,005)	0.63% (11/1,759)	1.37% (11/804)	2.83% (32/1,131)
	**3rd tertile**	1.43% (6/419)	2.18% (22/1,009)	4.23% (36/852)	4.43% (103/2,323)

Supplementary Table 12 Univariable and multivariable adjusted hazard ratios for the different aortic features to estimate all-cause mortality

All-cause mortality					
		**No patients (events)**	**Univariable HR (95% CI)**	**Multivariable HR* (95% CI)**	**Multivariable HR** (95% CI)**
**Diameter**	< 4.5cm	24,490 (1,697)	Reference	Reference	NA
	≥ 4.5cm	280 (38)	2.06 (1.49-2.83, p<.001)	2.16 (1.32-3.52, p=0.002)	NA
**Volume**	<210ml	12,385 (642)	Reference	Reference	Reference
	≥210ml	12,385 (1,093)	1.74 (1.58-1.92, p<.001)	1.44 (1.14-1.83, p=0.002)	1.41 (1.11-1.79, p=0.004)
**Calcifications**	1st tertile	8,257 (326)	Reference	Reference	Reference
	2nd tertile	8,257 (471)	1.46 (1.27-1.69, p<.001)	1.17 (0.85-1.61, p=0.35)	1.17 (0.85-1.61, p=0.34)
	3rd tertile	8,256 (938)	3.03 (2.67-3.44, p<.001)	2.58 (1.92-3.48, p<.001)	2.57 (1.91-3.47, p<.001)

*adjusted for adjusted for race, age at screening, sex, BMI, smoking status (former vs. current), prevalent diabetes, prevalent hypertension, history of myocardial infarction, and stroke; **adjusted for the same covariates plus maximum diameter

HR=hazard ratio; CI=confidence interval, BMI=body mass index

Supplementary Table 13 Univariable and multivariable adjusted hazard ratios for the different aortic features and coronary artery calcification to estimate all-cause mortality

All-cause mortality					
		**No patients (events)**	**Multivariable HR* (95% CI)**	**Multivariable HR** (95% CI)**	**Multivariable HR*** (95% CI)**
**Diameter**	< 4.5cm	13,741 (975)	Reference	Reference	Reference
	≥ 4.5cm	157 (20)	1.29 (0.83-2.02, p=0.26)	1.24 (0.79-1.94, p=0.34)	1.22 (0.78-1.90, p=0.38)
**Volume**	<210ml	6,988 (377)	Reference	Reference	Reference
	≥210ml	8,023 (618)	1.16 (0.99-1.36, p=0.05)	1.12 (0.96-2.01, p=0.14)	1.09 (0.93-1.27, p=0.29)
**Calcifications**	1st tertile	4,596 (172)	Reference	Reference	Reference
	2nd tertile	4,699 (284)	1.42 (1.17-1.72, p<.001)	1.33 (1.09-1.62, p=0.004)	1.33 (1.09-1.62, p=0.004)
	3rd tertile	4,603 (539)	2.22 (1.84-2.69, p<.001)	1.93 (1.58-2.36, p<.001)	1.92 (1.57-2.34, p<.001)

*adjusted for adjusted for race, age at screening, sex, BMI, smoking status (former vs. current), prevalent diabetes, history of myocardial infarction, arterial hypertension and stroke; **adjusted for the same covariates plus coronary artery calcifications; *** adjusted for the same covariates plus coronary artery calcification and all aortic features simultaneously

HR=hazard ratio; CI=confidence interval, BMI=body mass index

Supplementary Figures

Consort diagram

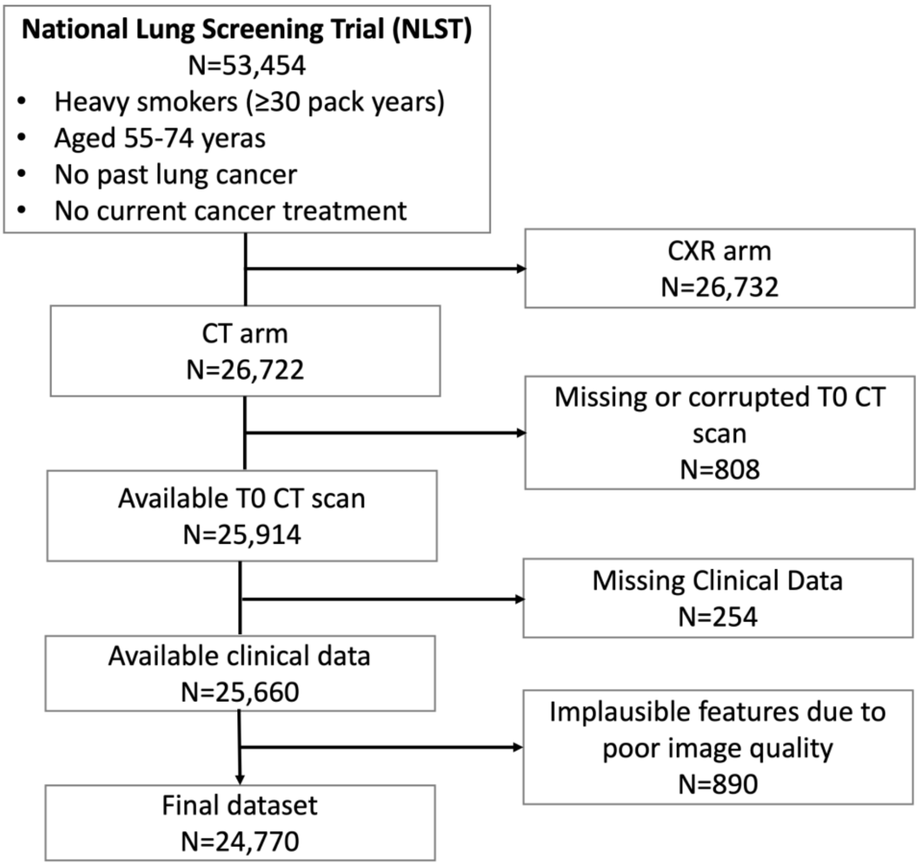

Supplementary Figure 1: Consort diagram

Performance of the deep learning framework

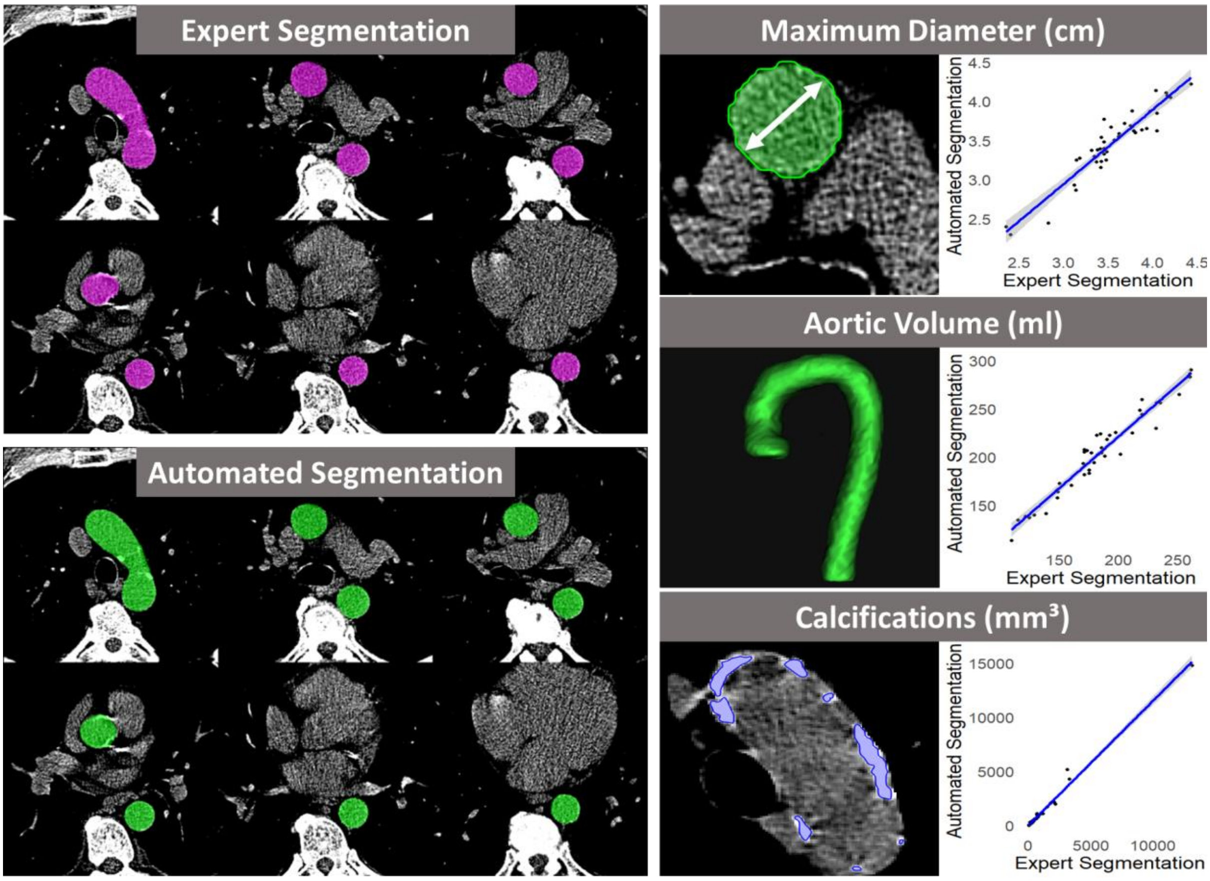

Supplementary Figure 2: Example case with manually generated expert segmentations (purple) and automatically generated deep learning segmentations (green) in a randomly selected participant of the test set. The right column depicts the aortic features extracted from the segmentation masks and the performance of the deep learning pipeline in the independent test data set.

Kaplan Meier survival analysis for the different aortic diameter cutoffs to estimate cardiovascular and all-cause mortality

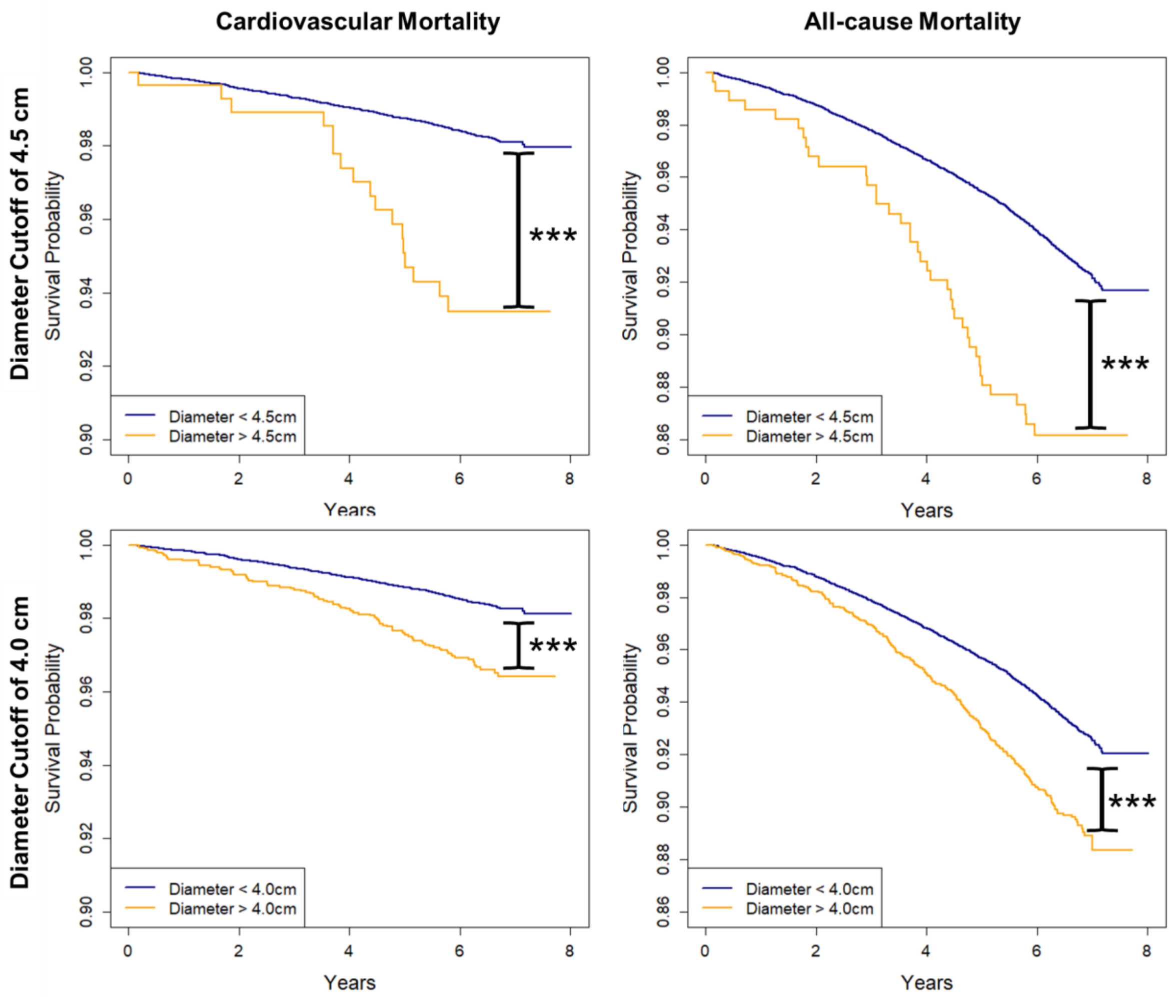

Supplementary Figure 3: Kaplan Meier survival analysis for different aortic diameter cutoffs to estimate cardiovascular mortality and all-cause mortality. Pairwise comparison of survival curves was performed using two-sided Log-Rank tests. P-values are adjusted for multiple comparisons using the Bonferroni-Holm method; *p<0.05; **p<0.01; ***p<0.001.

Kaplan Meier survival analysis for the different aortic features to estimate cardiovascular mortality stratified by sex

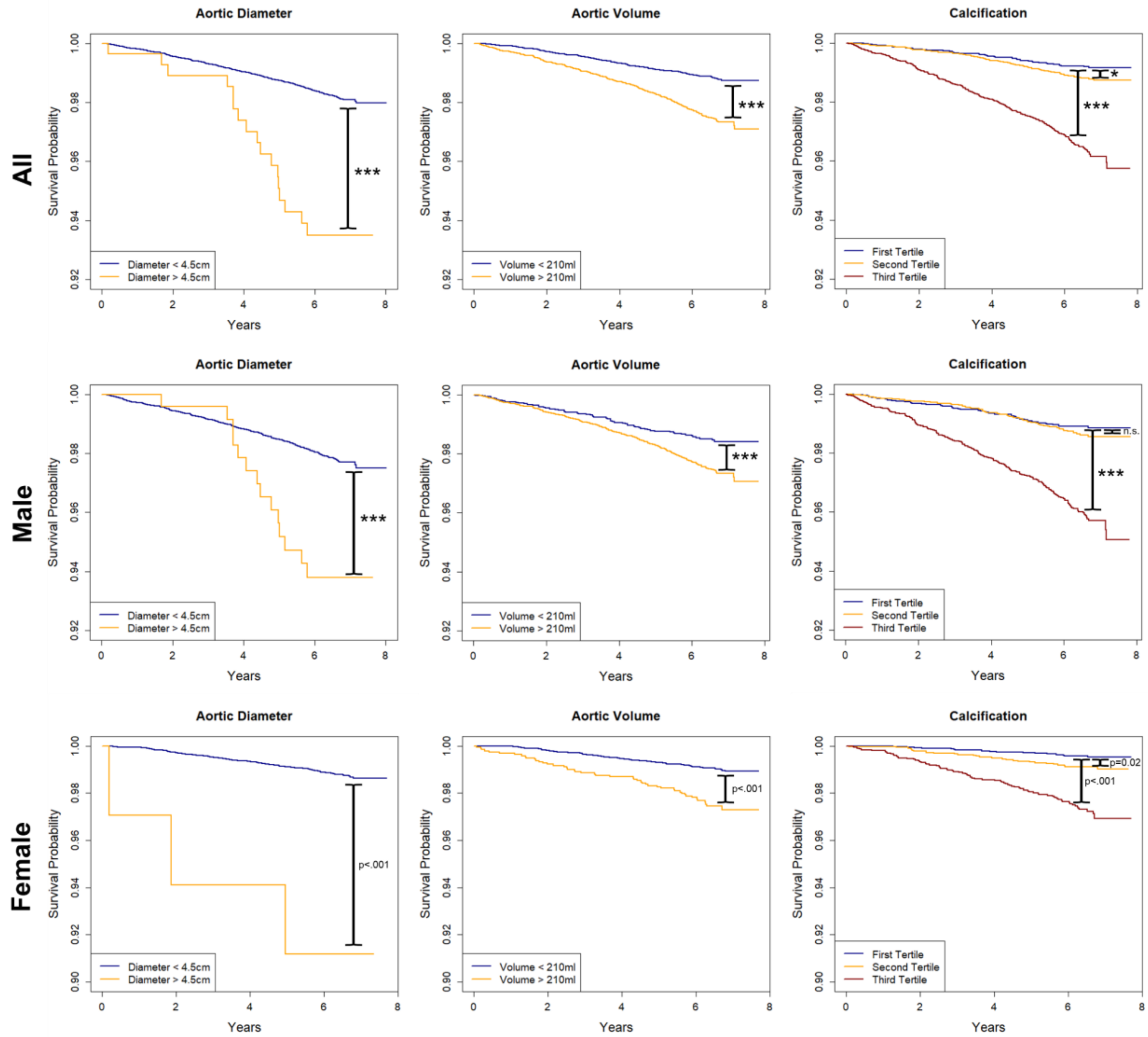

Supplementary Figure 4: Kaplan Meier survival analysis for the different aortic features to estimate cardiovascular mortality in **a**) the entire cohort, **b**) male and **c**) female participants. Kaplan Meier curves show worse outcomes for higher risk groups for all evaluated features. Pairwise comparison of survival curves was performed using two-sided Log-Rank tests. P-values are adjusted for multiple comparisons using the Bonferroni-Holm method; *p<0.05; **p<0.01; ***p<0.001.

Kaplan Meier survival analysis for the different aortic features to estimate cardiovascular mortality stratified by age

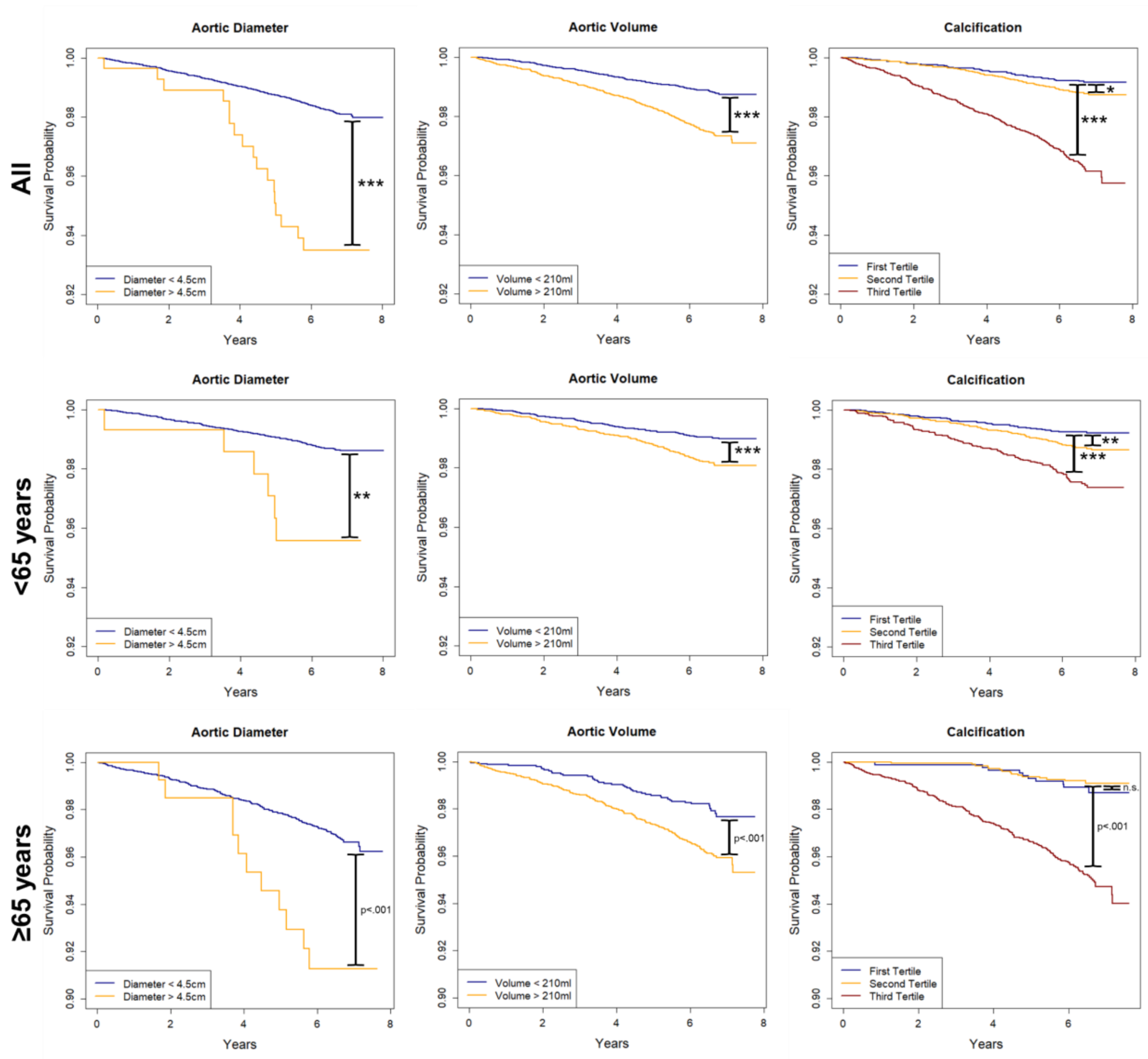

Supplementary Figure 5: Kaplan Meier survival analysis for the different aortic features to estimate cardiovascular mortality for **a**) the entire cohort, **b**) younger (<65-years-old) and **c**) older (≥65-years-old) participants. Kaplan Meier curves show worse outcomes for higher risk groups for all evaluated features. Pairwise comparison of survival curves was performed using two-sided Log-Rank tests. P-values are adjusted for multiple comparisons using the Bonferroni-Holm method; *p<0.05; **p<0.01; ***p<0.001.

Kaplan Meier survival analysis for the different aortic features to estimate cardiovascular mortality stratified by hypertension

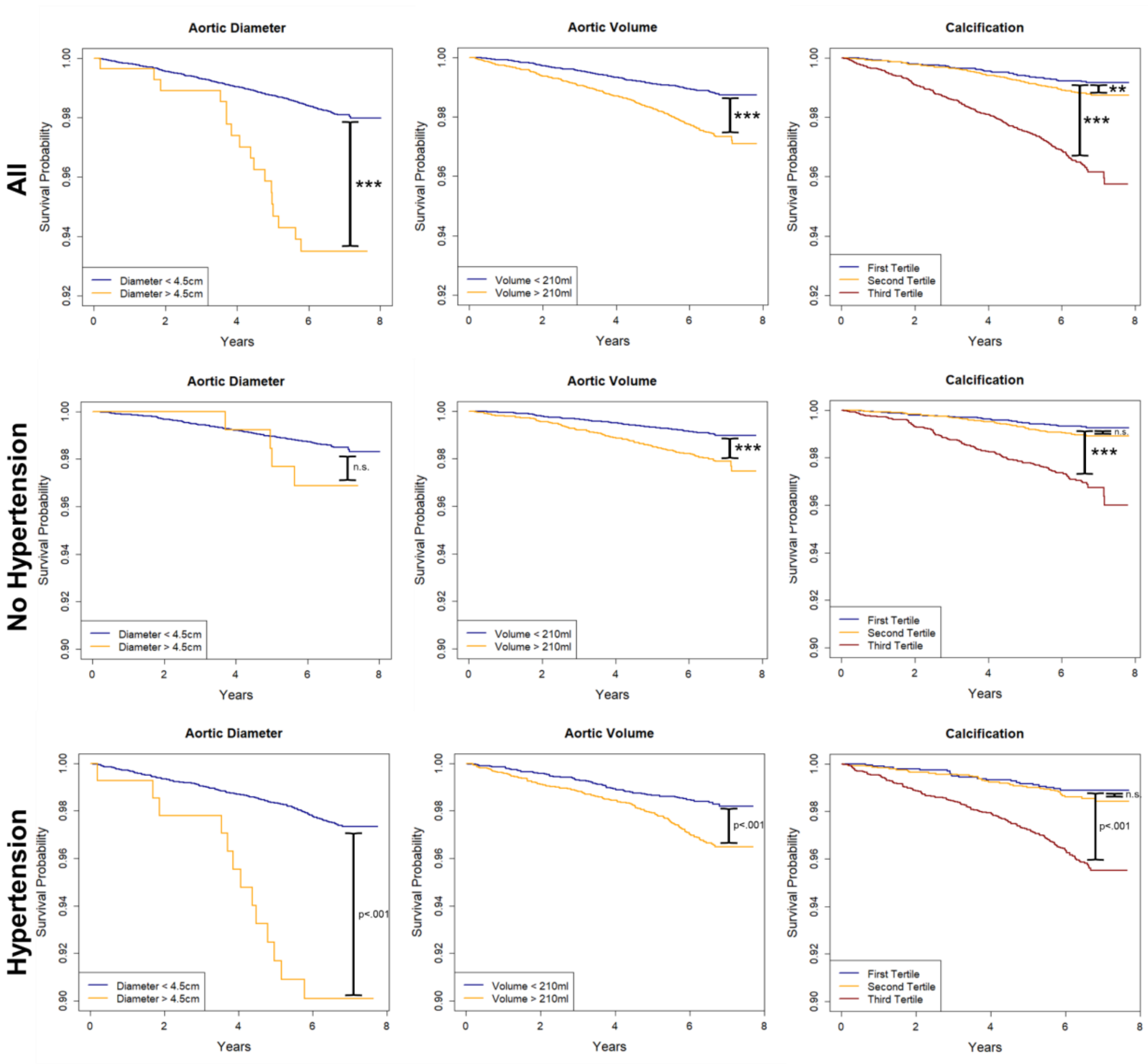

Supplementary Figure 6: Kaplan Meier survival analysis for the different aortic features and cardiovascular mortality for **a**) the entire cohort, **b**) participants without and **c**) with prevalent hypertension. Kaplan Meier curves show worse outcomes for higher risk groups for all evaluated features. Pairwise comparison of survival curves was performed using two-sided Log-Rank tests. P-values are adjusted for multiple comparisons using the Bonferroni-Holm method; *p<0.05; **p<0.01; ***p<0.001.

Kaplan Meier survival analysis for the different aortic features to estimate cardiovascular mortality in individuals without history of cardiovascular disease

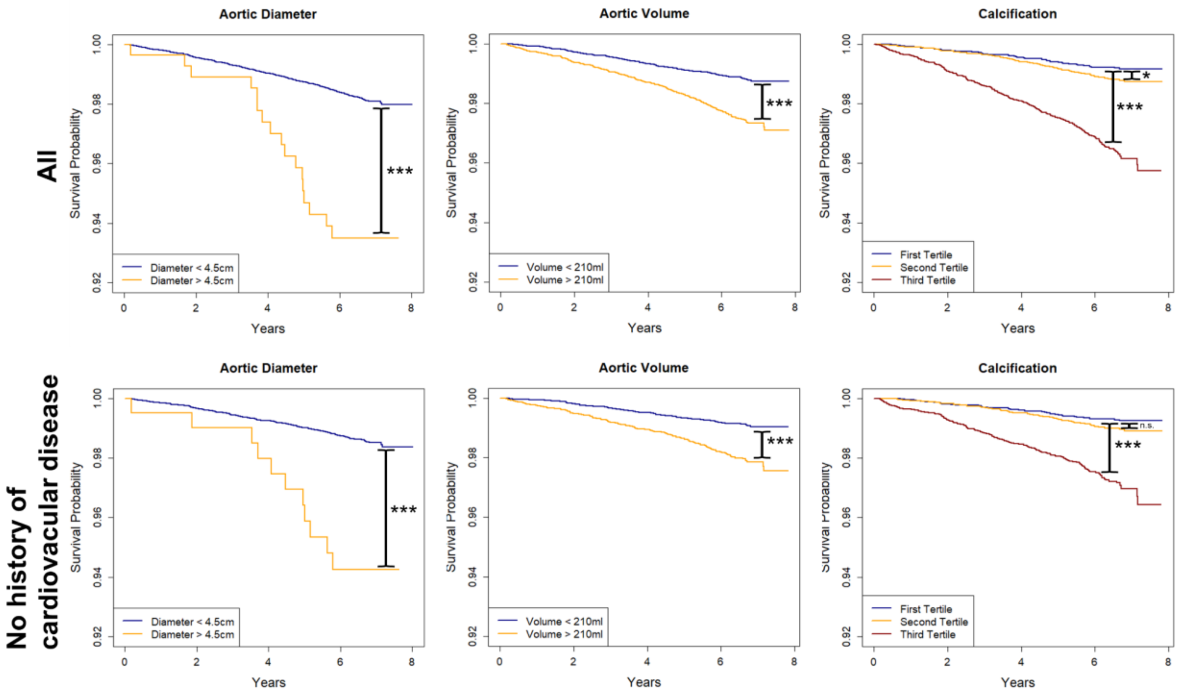

Supplementary Figure 7: Kaplan Meier survival analysis for the different aortic features to estimate cardiovascular mortality for **a**) the entire cohort and **b**) participants without history of cardiovascular disease (defined as no prior stroke or heart disease). Kaplan Meier curves show worse outcomes for higher risk groups for all evaluated features. Pairwise comparison of survival curves was performed using two-sided Log-Rank tests. P-values are adjusted for multiple comparisons using the Bonferroni-Holm method; *p<0.05; **p<0.01; ***p<0.001.

Kaplan Meier survival analysis for the different aortic features to estimate all-cause mortality

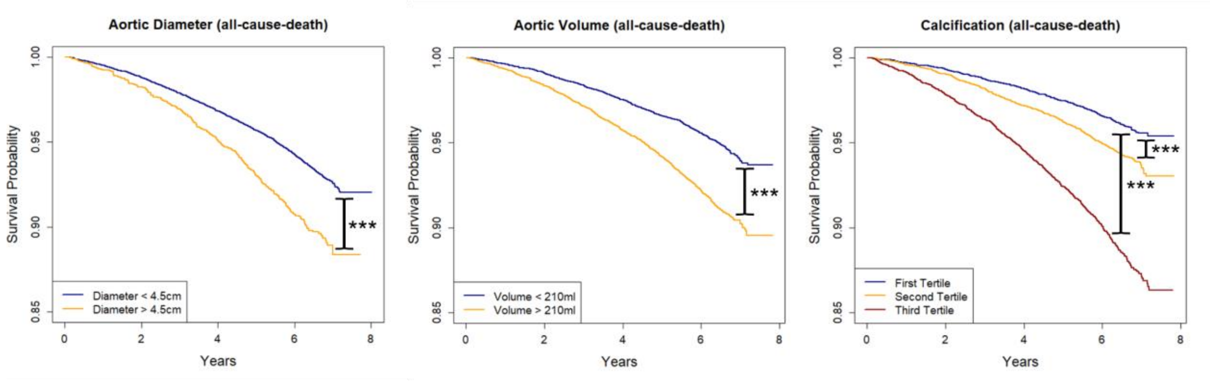

Supplementary Figure 8: Kaplan Meier survival analysis for the different aortic features to estimate all-cause mortality. Curves show worse outcomes for higher risk groups for all evaluated features. Pairwise comparison of survival curves was performed using two-sided Log-Rank tests. P-values are adjusted for multiple comparisons using the Bonferroni-Holm method; *p<0.05; **p<0.01; ***p<0.001.

Kaplan Meier survival analysis for the different aortic features to estimate all-cause mortality stratified by sex

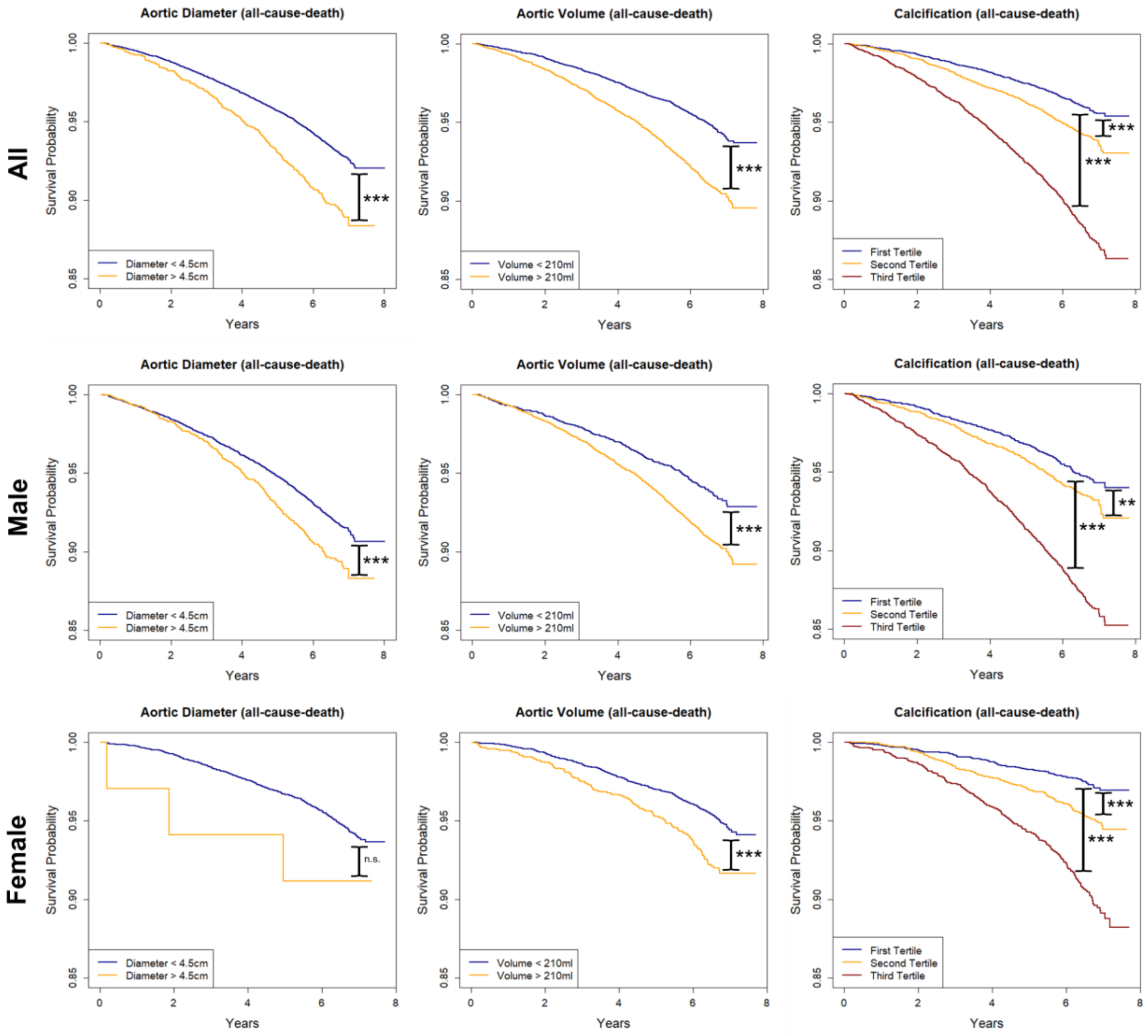

Supplementary Figure 9: Kaplan Meier survival analysis for the different aortic features to estimate all-cause mortality for **a**) the entire cohort, **b**) male and **c**) female participants. Kaplan Meier curves show worse outcomes for higher risk groups for all evaluated features. Pairwise comparison of survival curves was performed using two-sided Log-Rank tests. P-values are adjusted for multiple comparisons using the Bonferroni-Holm method; *$p<0.05$; **$p<0.01$; ***$p<0.001$.

Kaplan Meier survival analysis for the different aortic features to estimate all-cause mortality stratified by age

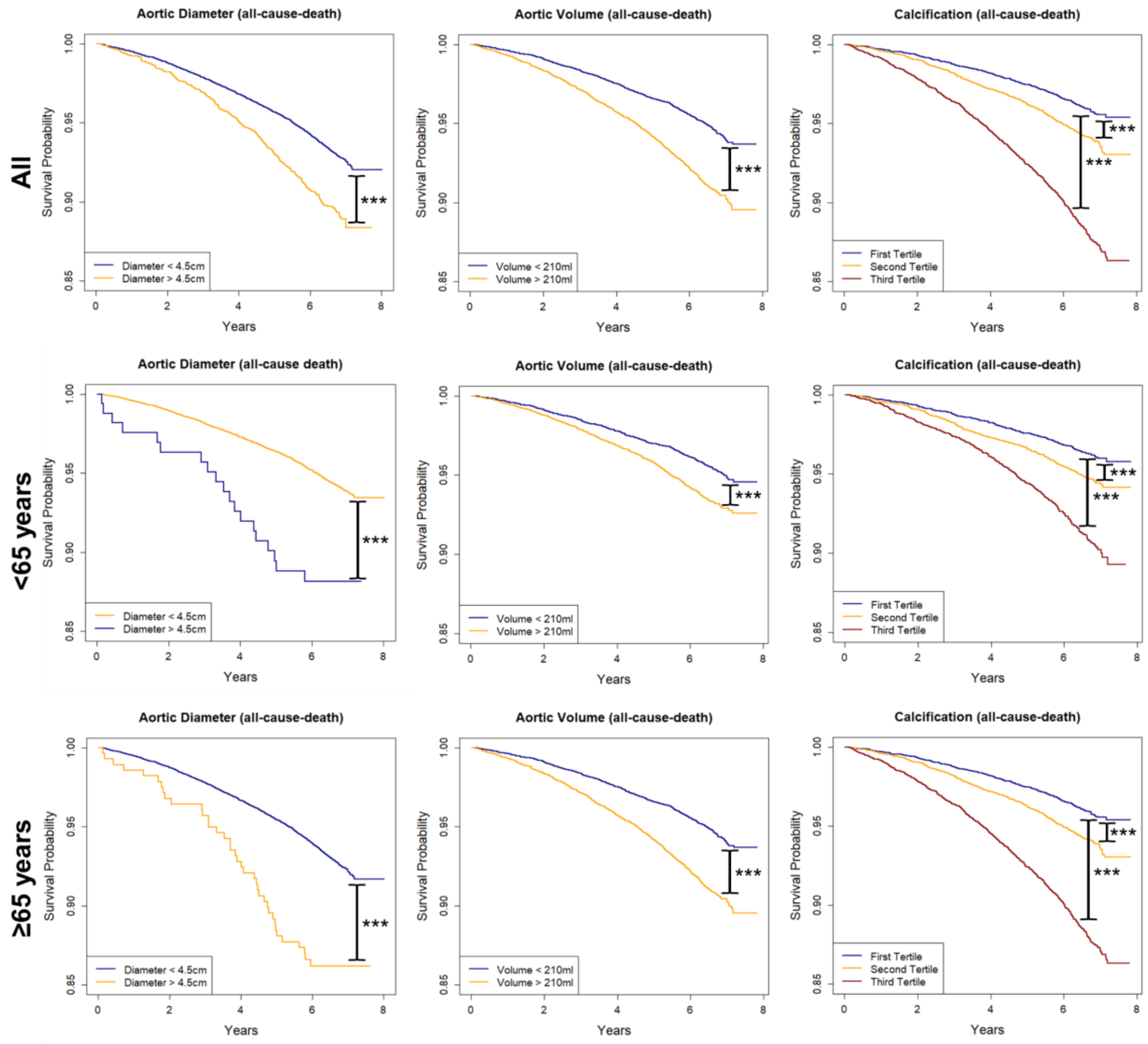

Supplementary Figure 10: Kaplan Meier survival analysis for the different aortic features to estimate all-cause mortality for **a**) the entire cohort, **b**) younger (<65-years-old) and **c**) older (≥65-years-old) participants. Kaplan Meier curves show worse outcomes for higher risk groups for all evaluated features. Pairwise comparison of survival curves was performed using two-sided Log-Rank tests. P-values are adjusted for multiple comparisons using the Bonferroni-Holm method; *p<0.05; **p<0.01; ***p<0.001.

Kaplan Meier survival analysis for the different aortic features to estimate all-cause mortality stratified by prevalent hypertension

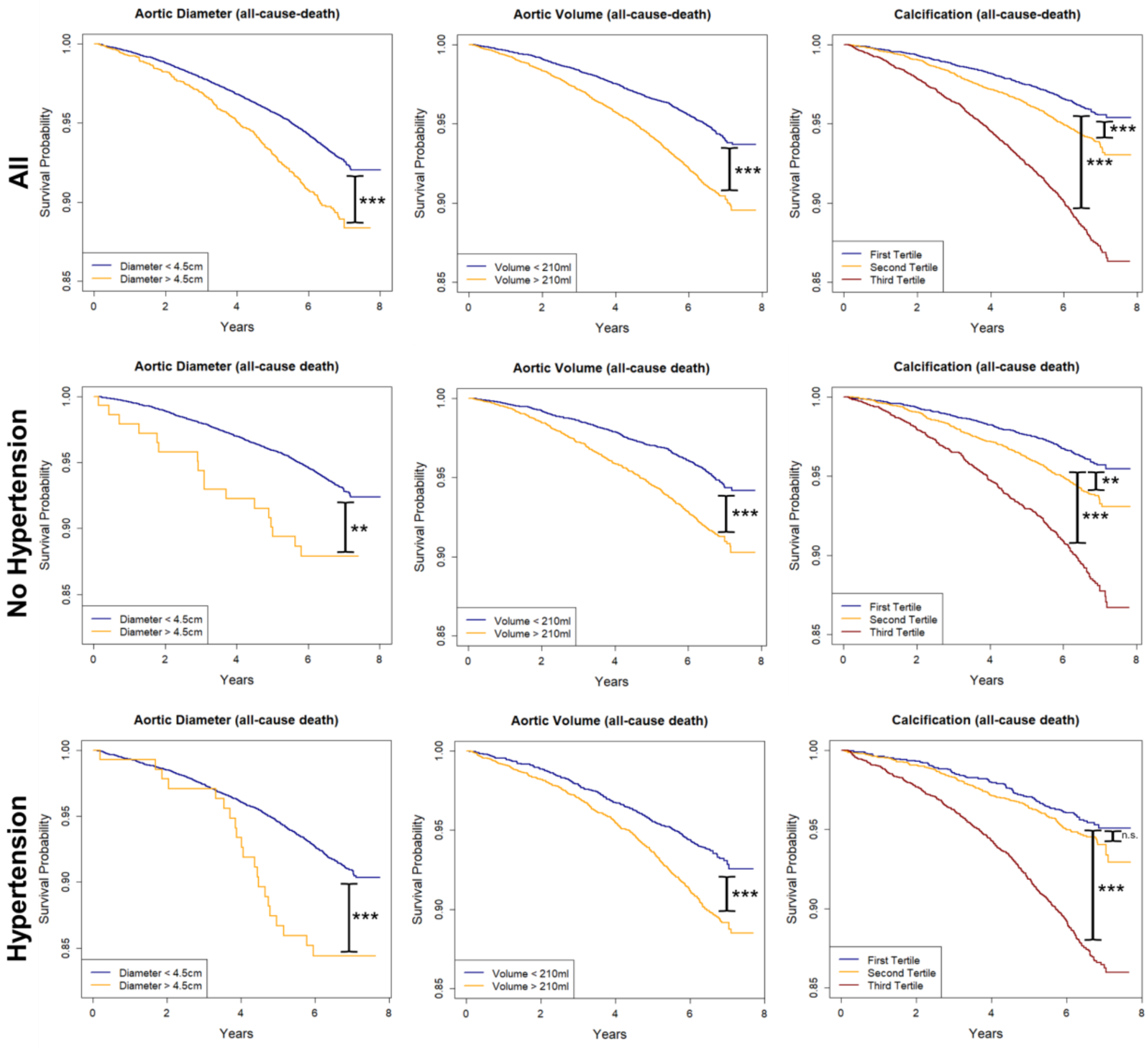

Supplementary Figure 11: Kaplan Meier survival analysis for the different aortic features to estimate all-cause mortality for **a**) the entire cohort, **b**) younger (<65-years-old) and **c**) older (≥65-years-old) participants. Kaplan Meier curves show worse outcomes for higher risk groups for all evaluated features. Pairwise comparison of survival curves was performed using two-sided Log-Rank tests. P-values are adjusted for multiple comparisons using the Bonferroni-Holm method; *p<0.05; **p<0.01; ***p<0.001.

Kaplan Meier survival analysis for the different aortic features to estimate cardiovascular mortality in individuals without history of cardiovascular disease

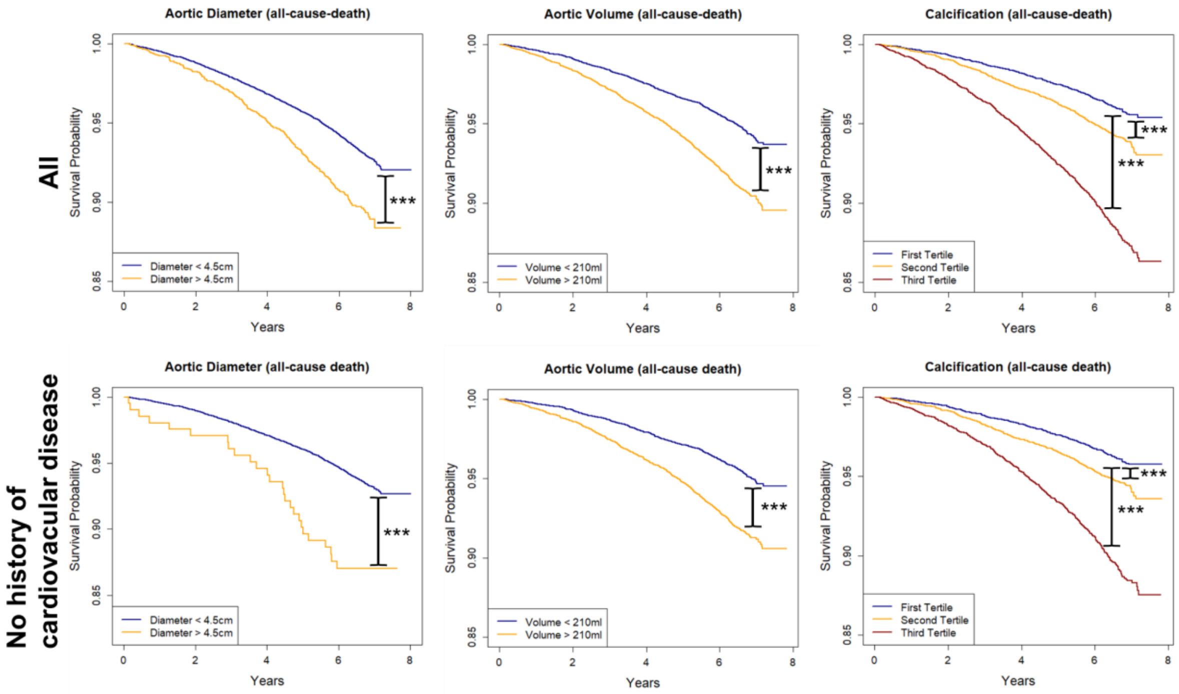

Supplementary Figure 12: Kaplan Meier survival analysis for the different aortic features to estimate cardiovascular mortality for **a**) the entire cohort and **b**) participants without history of cardiovascular disease (defined as no prior stroke or heart disease). Kaplan Meier curves show worse outcomes for higher risk groups for all evaluated features. Pairwise comparison of survival curves was performed using two-sided Log-Rank tests. P-values are adjusted for multiple comparisons using the Bonferroni-Holm method; *p<0.05; **p<0.01; ***p<0.001.
